# Investigating the influence of collagen cross-linking on mechanical properties of thoracic aortic tissue

**DOI:** 10.3389/fbioe.2024.1305128

**Published:** 2024-02-27

**Authors:** Chung Won Lee, Chiseung Lee, Seungik Baek, Emrah Akkoyun, Dongman Ryu

**Affiliations:** ^1^ Department of Thoracic and Cardiovascular Surgery, School of Medicine, Pusan National University, Busan, Republic of Korea; ^2^ Biomedical Research Institute, Pusan National University Hospital, Busan, Republic of Korea; ^3^ Department of Convergence Medicine and Biomedical Engineering, School of Medicine, Pusan National University, Busan, Republic of Korea; ^4^ Department of Mechanical Engineering, Michigan State University, East Lansing, MI, United States; ^5^ TÜBITAK-ULAKBIM, Turkish Academic Network and Information Center, Ankara, Türkiye; ^6^ Medical Research Institute, Pusan National University, Busan, Republic of Korea

**Keywords:** thoracic aorta, uniaxial tensile test, cross-linking, regional difference, ultimate mechanical characteristic

## Abstract

Vascular diseases, such as abdominal aortic aneurysms, are associated with tissue degeneration of the aortic wall, resulting in variations in mechanical properties, such as tissue ultimate stress and a high slope. Variations in the mechanical properties of tissues may be associated with an increase in the number of collagen cross-links. Understanding the effect of collagen cross-linking on tissue mechanical properties can significantly aid in predicting diseased aortic tissue rupture and improve the clarity of decisions regarding surgical procedures. Therefore, this study focused on increasing the density of the aortic tissue through cross-linking and investigating the mechanical properties of the thoracic aortic tissue in relation to density. Uniaxial tensile tests were conducted on the porcine thoracic aorta in four test regions (anterior, posterior, distal, and proximal), two loading directions (circumferential and longitudinal), and density increase rates (0%–12%). As a result, the PPC (Posterior/Proximal/Circumferential) group experienced a higher ultimate stress than the PDC (Posterior/Distal/Circumferential) group. However, this relationship reversed when the specimen density exceeded 3%. In addition, the ultimate stress of the ADC (Anterior/Distal/Circumferential) and PPC group was greater than that of the APC (Anterior/Proximal/Circumferential) group, while these findings were reversed when the specimen density exceeded 6% and 9%, respectively. Finally, the high slope of the PDL (Posterior/Distal/Longitudinal) group was lower than that of the ADL (Anterior/Distal/Longitudinal) group, but the high slope of the PDL group appeared larger due to the stabilization treatment. This highlights the potential impact of density variations on the mechanical properties of specific specimen groups.

## 1 Introduction

Collagen and elastin are the predominant fibrous proteins in the arterial wall, and they play an important role in mechanical resistance, tensile strength, and elasticity (Boucek, 1998; Carmo et al., 2002; Sokolis et al., 2006). Elastin is essential for expansibility, whereas collagen contributes significantly to aortic stiffness and tensile strength. Consequently, they play an important role in distinguishing “soft” and “hard” connective tissue (Vogel, 1978; Haut, 1985; Sokolis et al., 2006). Moreover, elastin can stretch beyond 300% of its original length but fails at relatively low stress levels (Dingemans et al., 2000). In contrast, collagen is less flexible than elastin, but can withstand significantly higher stress loads, thereby significantly contributing to aortic strength (Lee and Kamm, 1994).

The mechanical properties of the arterial wall vary depending on age and the presence or absence of disease. Numerous studies have demonstrated that hypertension, which is considered a risk factor for diseases, such as cerebral infarction and ischemic heart failure, influences the dimensions and characteristics of the arterial walls (Fung, 1993; Hayashi et al., 1996; Humphrey, 2002; Hayashi and Naiki, 2003; Holzapfel, 2003). In other instances, aortic aneurysms are stiffer and less expanded than those of the nonaneurysmal abdominal aorta (Xiong et al., 2008). According to Carmo et al. (Carmo et al., 2002) and Pichamuthu et al. (Pichamuthu et al., 2013), the collagen and elastin contents of aneurysmal samples differ from those of non-aneurysmal samples, and the tensile strength of ascending aortic aneurysm samples differs from that of non-aortic aneurysm samples. In addition to these diseases, numerous studies have been conducted to analyze changes in mechanical properties and collagen and elastin content with age (Roach and Burton, 1957; Wolinsky and Glagov, 1964; Brüel and Oxlund, 1996). Nonetheless, because there was no difference in the collagen-to-elastin content ratio with age, an extensive histological study found no agreement in age-related changes in arterial wall elasticity and composition (Brüel and Oxlund, 1996; Hayashi and Hirayama, 2017). This suggests that factors other than collagen and elastin play a significant role in the changes in the mechanical properties of the arteries. Collagen cross-linking is an example of this phenomenon.

Collagen cross-linking increases as aortic collagen is remodeled during postnatal maturation. This increase in collagen cross-linking may serve to increase aortic tissue tensile stress by strengthening and stiffening the aortic wall (Brüel and Oxlund, 1996; Wells et al., 1998; Cantini et al., 2001; Barodka et al., 2011; Uzel and Buehler, 2011; Hayashi and Hirayama, 2017). Moreover, although the mechanisms of arterial dilatation and aneurysm formation are unknown, it has been suggested that elastin and collagen degradation play vital roles (Carmo et al., 2002; Villard et al., 2017).

Studies to understand the mechanical properties of arterial tissue as well as studies to understand the increase in collagen cross-links in arterial tissue in arterial disease should be conducted to clearly investigate the mechanism of arterial disease. Several studies have been conducted to identify some characteristics of arterial tissues in light of the aforementioned collagen cross-links; however, these are still insufficient. Therefore, in this study, we aimed to examine and quantitatively evaluate the mechanical properties of thoracic aortic tissue while considering collagen cross-linking. In the first step of inducing collagen cross-linking in the thoracic aortic tissue, the change in density of the aortic tissue was investigated to examine the influence of collagen cross-linking on the mechanical behavior of the aortic tissue. In particular, aortic tissue is treated with NaBH4 to reduce immature and unstable cross-links and increase stabilized collagen cross-links (ISO, 2017). Second, because the mechanical properties differ depending on location, the thoracic aortic tissue was divided into four zones. Finally, a uniaxial tensile test was performed to determine the collagen failure characteristics of the thoracic aortic tissue considering collagen cross-linking, and the experimental results were quantitatively evaluated.

## 2 Methods

### 2.1 Tissue harvest and sample preparation

**FIGURE 1:**
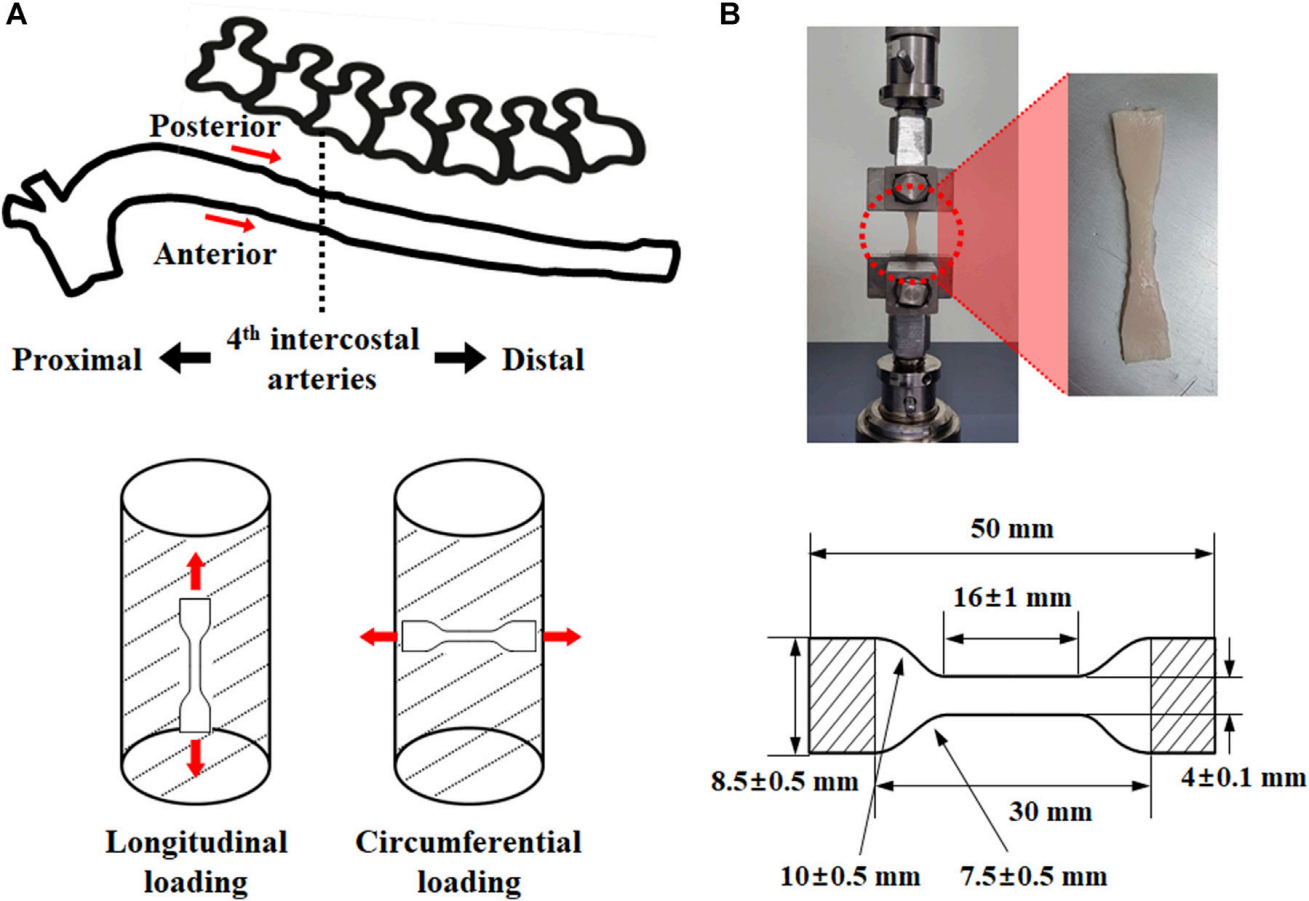
Test specimen extraction **(A)** position (anterior side of proximal: AP, posterior side of proximal: PP, anterior side of distal: AD, posterior side of distal: PD) and load direction (longitudinal direction: L, circumferential direction: C), as well as **(B)** specimen specification.

Thoracic aortic tissue was collected from six-month-old porcine aortic tissue. They were used for experiments immediately after the loose connective tissue of the adventitia was removed. A tensile specimen was prepared using the extracted aorta in accordance with ISO 37 Type 3 (Figure 1) (Davison, 1989). A Vernier caliper was used to measure the thickness of the specimens twice at three different points. In addition, the thoracic aorta was divided into eight sets based on the direction and location of the load to investigate its mechanical behavior. The specimen was prepared for the regional classification criterion by dividing it into proximal and distal parts based on the fourth intercostal artery. Some tissue samples were tested immediately after harvesting, whereas others were frozen in saline and subsequently tested at a later time.

### 2.2 Stabilization treatment

The prepared tensile specimens were placed in a saline-filled cylinder, and the increase in saline level and weight (cylinder, saline, and specimen) were measured. After NaBH4 treatment, a tensile specimen was placed in a saline-filled cylinder, and the increasing level and saline weight (cylinder, saline, and specimen) were measured. The rate of density increase due to cross-linking was calculated based on the increased saline level and weight (cylinder, saline solution, and specimen). In accordance with the calculated density increase rate, a range of 3% was established and denoted by a number following the name of the sample. For instance, PPC1 refers to a specimen that has not undergone the stabilization treatment. In addition, PPC2 refers to the group of specimens whose density increased from 0.1% to 3% in the specimens subjected to the stabilization treatment, PPC3 from 3% to 6%, PPC4 from 6% to 9%, and PPC5 from 9% to 12%.

NaBH4 treatment can either reduce or significantly increase the density of immature cross-links (Wells et al., 1998; Hayashi and Hirayama, 2017). This may reduce or increase the density of the aortic tissue following stabilization treatment. Therefore, in this study, a mechanical performance evaluation was performed only for specimens with increased density, and the relative contribution of the mechanical behavior to the density increase rate was examined. Tensile specimens were stabilized by four cycles of constant stirring for 15 min in 0.1 mg/mL NaBH4/borate buffer solution (pH = 9.0) at 4°C (Wells et al., 1998). An electronic overhead stirrer (Global Lab, SL 4000) was used for constant stirring at 250 RPM.

### 2.3 Test apparatus and uniaxial tensile test

A 500 N EZ-Test load cell (Shimadzu) and a universal testing machine were used for the uniaxial tensile test, and the experimental environment was maintained at room temperature. To minimize the slip, the end of the tensile specimen was fixed with sandpaper and a fixing device. In addition, before the uniaxial tensile test, 10 cycles were performed on 10% of the specimen length at a stretch rate of 3 mm/min to confirm constant stress and stretch of the specimen (Raghavan et al., 1996; Wang et al., 2001). The uniaxial tensile test speed was controlled at 3 mm/min, and physiological saline was used to maintain the material specimen wetness.

### 2.4 Data analysis

**FIGURE 2:**
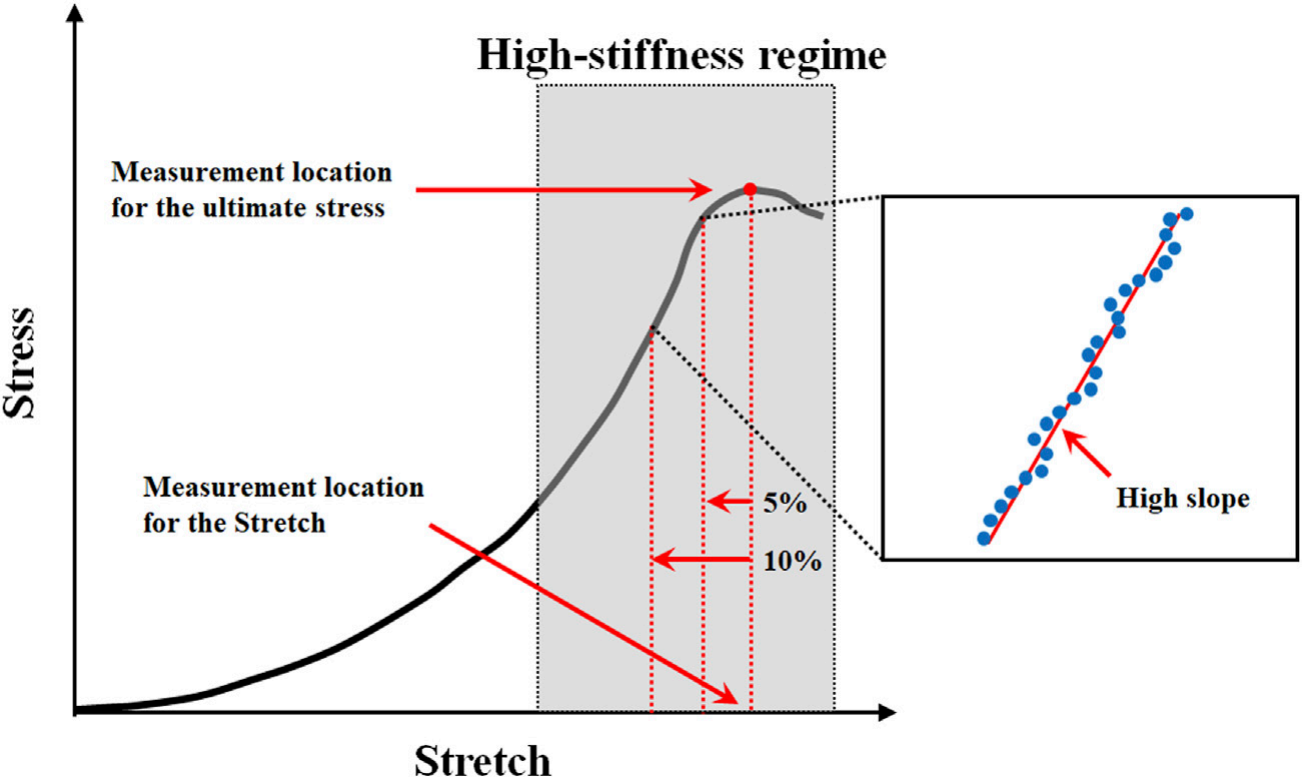
Measurement location for the ultimate stress, stretch, and high slope in typical stress-stretch curve of aortic tissue undergoing tensile testing.

The ultimate stress, stretch, and high slope were investigated in this study using the stress-stretch curve extracted from a uniaxial tensile test based on the density of the material specimen, extraction location, and loading condition. The measurement locations for the ultimate stress, stretch, and high slope in the stress-stretch curve are shown in Figure 2, where the stretch was measured at the point where the ultimate stress appeared. Furthermore, it is extremely difficult to measure the high slopes of nonlinear curves. As a result, to solve this problem, the high-slope region is defined in this study with the same calculation conditions for calculating the high slope in all curves (Figure 2). A straight line was derived using the least-squares method by extracting the stress-stretch data in the high-slope region, which was located between 5% and 10% in the initial direction from the ultimate stress point. At this point, the slope is referred to as the high slope of the stress-stretch curve. The interval between 5% and 10% corresponds to the portion where the coefficient of determination (R2) between the value obtained from the experimental result and the derived straight line is equal to or greater than 0.999.

### 2.5 Statistical analysis

The Shapiro-Wilk test was used to examine the normal distribution of the ultimate stress, stretch, and high slope in all regions. As a result, the Student's t-test (if data normality is satisfied) and Mann-Whitney U test (if data normality is not satisfied) were used to compare the ultimate stress, stretch, and high slope in density variation and different regions. Student’s t-test and Mann-Whitney U test were also used to examine differences in outcome values between groups in relation to the loading direction. The median and interquartile range (25%–75%) are displayed in the comparison graph of the ultimate stress, stretch, and high slope according to the density variation, sample extraction position, and loading direction. Finally, Pearson and Spearman rank correlation studies were performed to determine the relationship between the high slope, ultimate stress, and stretch based on the density variation and sample extraction position. SPSS Statistics was used for statistical analysis.

## 3 Results

**FIGURE 3:**
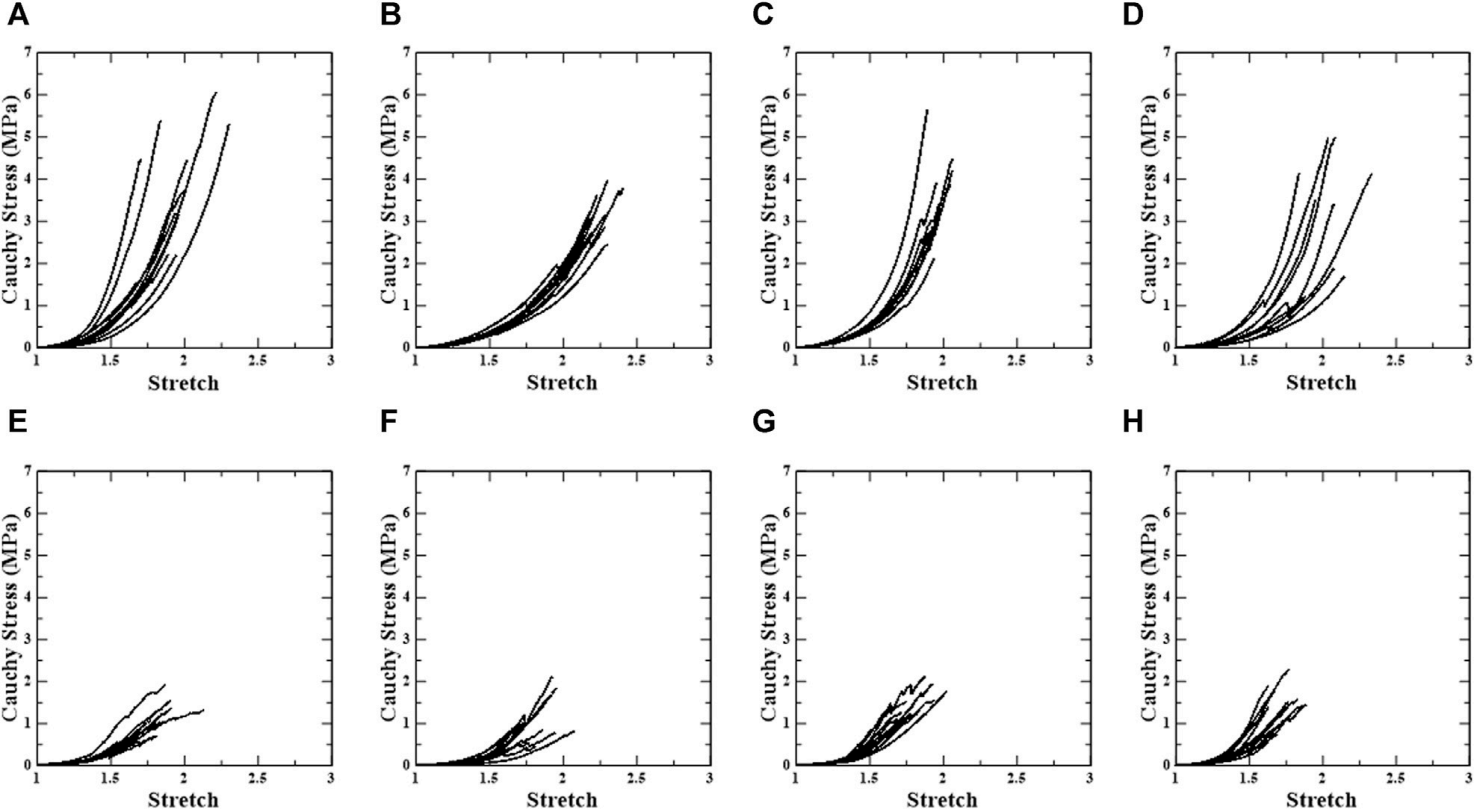
Uniaxial stress-stretch curve of test regions. First row shows circumferential specimens [**(A)** PPC, **(B)** APC, **(C)** PDC, and **(D)** ADC], and second row shows longitudinal specimens [**(E)** PPL, **(F)** APL, **(G)** PDL, and **(H)** ADL].

In this study, 124 samples of porcine thoracic aorta were obtained for uniaxial tensile tests with different extraction locations, loading directions, and density conditions, and 478 specimens (PPC: n = 62, APC: n = 62, PDC: n = 59, ADC: n = 53, PPL: n = 62, APL: n = 60, PDL: n = 61, and ADL: n = 59) were obtained from 124 samples. Experimental results (n = 38) in which slipping of the specimen occurred in the grip area or the fracture point did not occur in the center were excluded. As a result, uniaxial stress-stretch curves (n = 440) for circumferential and longitudinal loading directions in the anterior, posterior, distal, and proximal areas of the porcine aorta were evaluated, as well as the material behavior based on the density variation of each specimen. The overall stress-stretch response was nonlinear, with regional variances. The ultimate stress ranges for material specimens under circumferential and longitudinal loading conditions are 0.72–6.41 MPa for stretches between 1.60 and 2.72 and 0.44–3.89 MPa for stretches between 1.50 and 2.23. Figure 3 shows representative stress-stretch curves for the extraction location and loading direction, excluding the density variation. The Supplementary Material S1 present graphs for the groups based on the extraction location, loading direction, and density (Supplementary Figures S1–4).

### 3.1 The influence of loading direction/test region/density on ultimate stress

**FIGURE 4:**
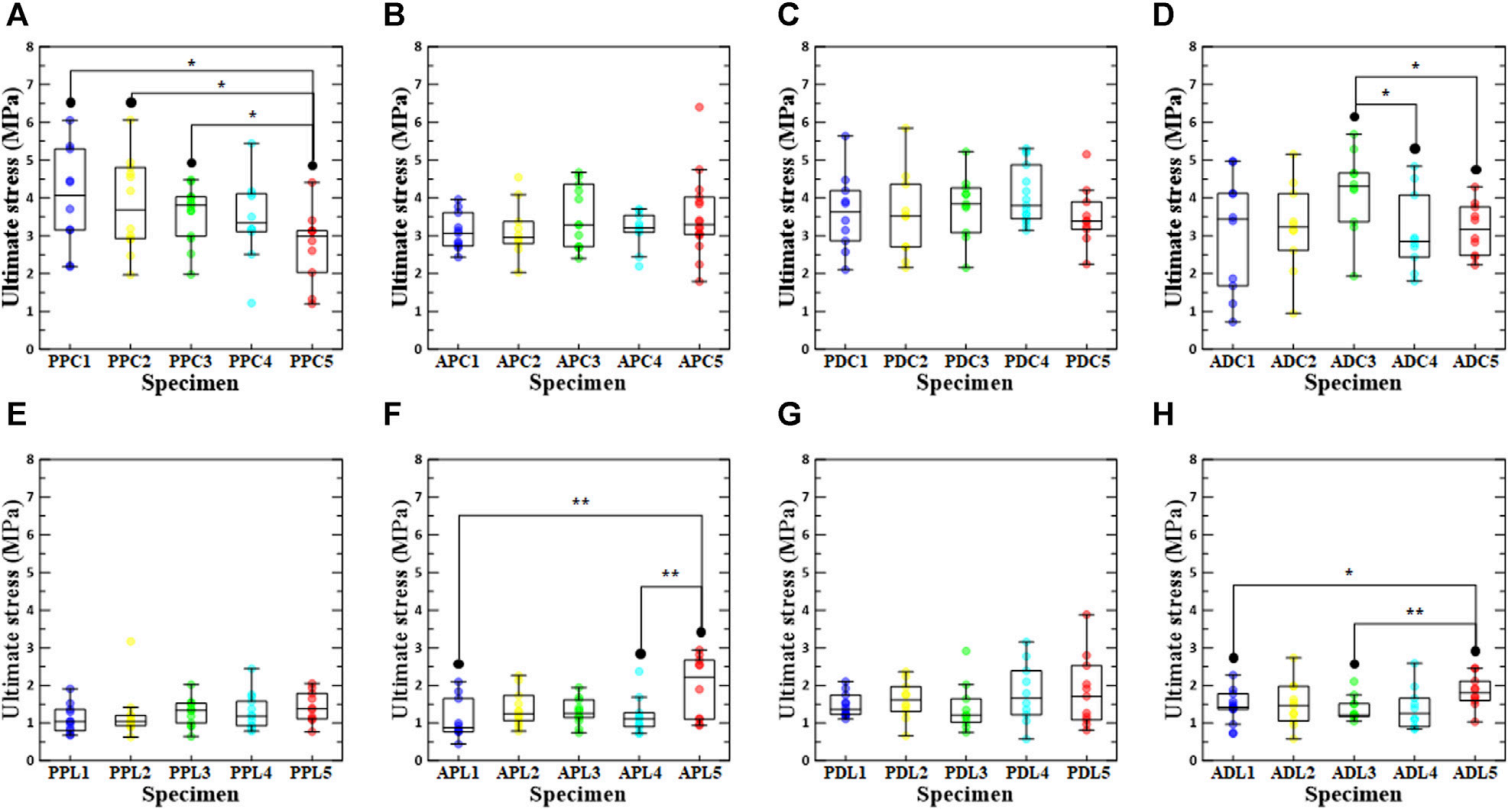
Ultimate stress according to density increase ratio and test region. The first row shows circumferential specimens [**(A)** PPC, **(B)** APC, **(C)** PDC, and **(D)** ADC], and the second row shows longitudinal specimens [**(E)** PPL, **(F)** APL, **(G)** PDL, and **(H)** ADL].

The ultimate stresses for each test were derived based on the test region, loading direction, and increase in density (Figure 4). The numbers in the specimen names in Figure 4 indicate the groups with an increase in density. For instance, PPC1 refers to a specimen that has not undergone the stabilization treatment. In addition, PPC2 refers to the group of specimens whose density increased from 0.1% to 3% in the specimens subjected to the stabilization treatment, PPC3 from 3% to 6%, PPC4 from 6% to 9%, and PPC5 from 9% to 12%. For specimens without stabilization treatment, the ultimate stress in the longitudinal direction is significantly lower than in the circumferential direction in the PPC1 vs. PPL1 (4.00 MPa (±0.424) vs. 1.11 MPa (±0.115), *p* < 0.001, respectively), APC1 vs. APL1 (3.11 MPa (±0.147) vs. 1.11 MPa (±0.173), *p* < 0.001, respectively), PDC1 vs. PDL1 (3.61 MPa (±0.325) vs. 1.46 MPa (±0.091), *p* < 0.001, respectively), ADC1 vs. ADL1 (3.05 MPa (±0.494) vs. 1.42 MPa (±0.131), *p* = 0.009, respectively). In addition, even for specimens with stabilization treatment, the ultimate stress was lower in the longitudinal direction than in the circumferential direction (Figure 4; Supplementary Table S1).

In the comparison results for ultimate stress according to the extraction location, PPC1 and ADL1 had the highest values among the groups without stabilization treatment, and PPC2/PDL2, ADC3/PPL3, PDC4/PDL4, and PDC5/APL5 had the highest values among the groups with stabilization treatment (0.1%–3%, 3%–6%, 6%–9%, and 9%–12%). Statistical analysis revealed significant differences between APC4 vs. PDC4 (3.15 MPa (±0.156) vs. 4.07 MPa (±0.196), *p* = 0.003, respectively), PDC4 vs. ADC4 (4.07 MPa (±0.196) vs. 3.10 MPa (±0.327), *p* = 0.013, respectively), PPL1 vs. PDL1 (1.11 MPa (±0.115) vs. 1.46 MPa (±0.091), *p* = 0.023, respectively), PPL2 vs. PDL2 (1.04 MPa (0.930, 1.21) vs. 1.62 MPa (1.31, 1.97), *p* = 0.024, respectively), APL4 vs. PDL4 (1.12 MPa (0.901, 1.28) vs. 1.66 MPa (1.22, 2.39), *p* = 0.041, respectively), and PPL5 vs. ADL5 (1.43 MPa (±0.131) vs. 1.84 MPa (±0.137), *p* = 0.047, respectively) (Supplementary Tables S2,3).

In the PPC groups, the ultimate stress of PPC1 was the highest in the comparison of ultimate stress according to density conditions, and the ultimate stress of PPC5 was significantly different from those of PPC1, PPC2, and PPC3. Among the ADC, APL, and ADL groups, ADC3, APL5, and ADL5 had the highest ultimate stresses, and there were significant differences between ADC5 and ADC3, ADC4 and ADC3, APL5 and APL1, APL5 and APL4, ADL5 and ADL1, and ADL5 and ADC3. The specimens with the highest ultimate stresses were APC5, PDC3, PPL5, and PDL5 among the APC, PDC, PPL, and PDL groups; however, there was no significant difference between the ultimate stresses according to the density condition. A comparison between the PPC and PDC specimens confirmed that the ultimate stress of the PDC specimen was greater than that of the PPC specimen at a density increase of 3% or more. In addition, a comparison between the APC and ADC specimens confirmed that the APC specimen had a higher ultimate stress than the ADC specimen, owing to a density increase rate of at least 9%. In the comparison between the PPC and APC specimens, it was only when the density increase rate was 9%–12% that the ultimate stress of the APC specimen was greater than that of the PPC specimen (Supplementary Tables S4, 5).

### 3.2 The influence of loading direction/test region/density on failure stretch

**FIGURE 5:**
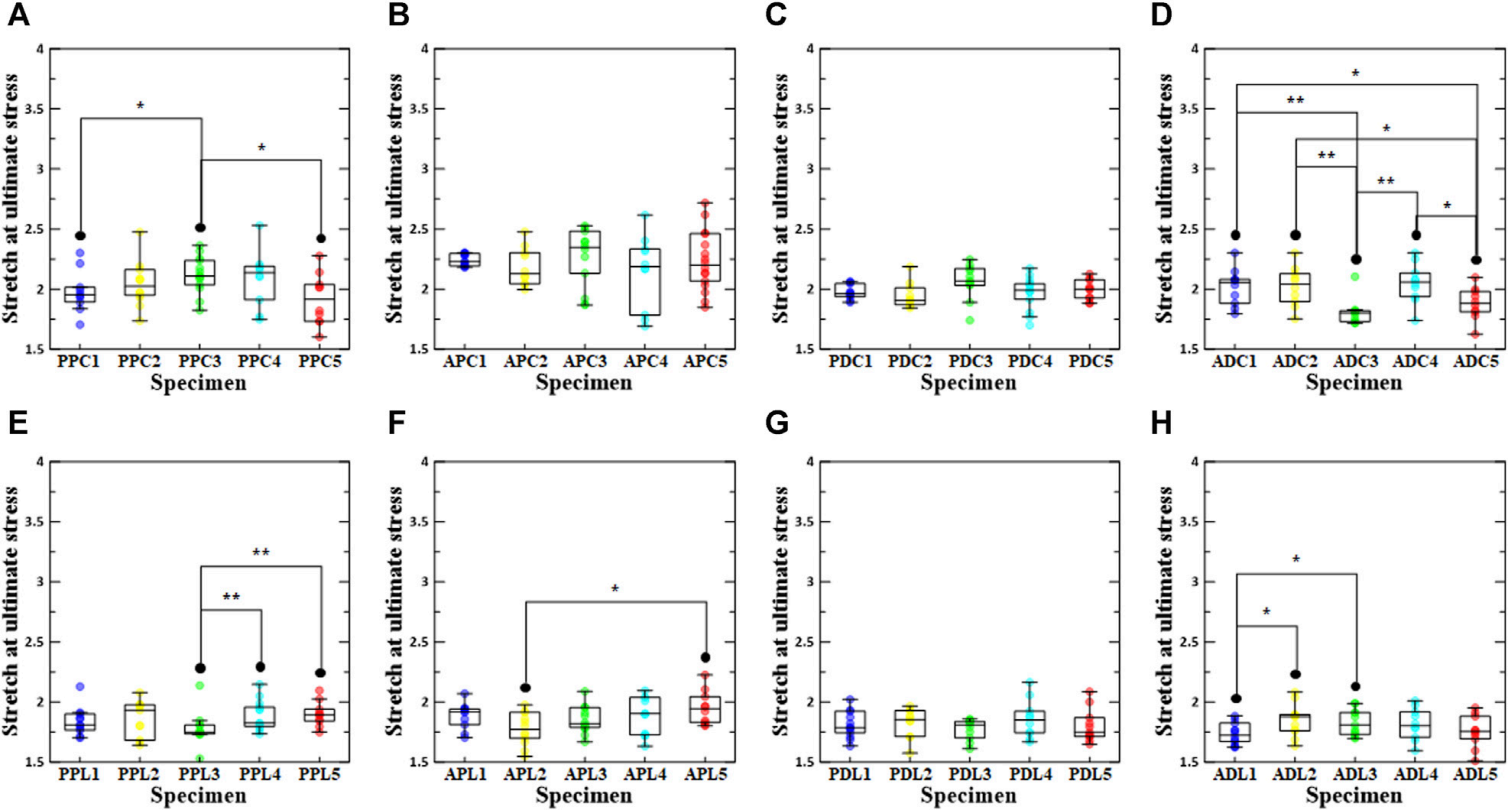
Stretch at ultimate stress according to density increase ratio and test region. The first row shows circumferential specimens [**(A)** PPC, **(B)** APC, **(C)** PDC, and **(D)** ADC], and the second row shows longitudinal specimens [**(E)** PPL, **(F)** APL, **(G)** PDL, and **(H)** ADL].

As shown in Figure 5, the failure stretch ratios at the ultimate stress point were determined for the test region, density condition, and loading direction. In conclusion, the failure stretch ratio was significantly lower in the longitudinal direction than in the circumferential direction for all specimens except ADC3. It was confirmed that there was a significant difference between the comparison results for the loading direction of all specimens, with the exception of the comparison results between ADC3 and ADL3, ADC5 and ADL5, PDC4 and PDL4, and PPC5 and PPL5, among the groups with stabilization treatment. In the regional comparative analysis of the circumferential loading direction, the APC groups had the highest median values regardless of the stabilization treatment (APC1:2.23 (2.19, 2.30), APC2:2.13 (2.05, 2.30), APC3:2.35 (2.13, 2.48), APC4:2.19 (1.78, 2.33), APC5:2.20 (2.07, 2.46)) (Supplementary Table S1). In the regional comparative analysis of the longitudinal loading direction, PPL2, the group with a density increase of 0.1%–3%, had the highest value among the groups with stabilization treatment, whereas the APL group had the highest value among the other groups (APL1:1.92 (1.81, 1.94), PPL2:1.93 (1.68, 1.98), APL3:1.82 (1.79, 1.95), APL4:1.90 (1.73, 2.04), APL5:1.94 (1.83, 2.04)). When comparing the failure stretch ratios based on the density conditions among the PPC groups, the failure stretch ratio of PPC4 was the highest, and the failure stretch ratio of PPC3 differed significantly from those of PPC1 and PPC5. In addition, ADC4, PPL2, APL5, and ADL2 had the highest stretch failure ratios among the ADC, PPL, APL, and ADL groups. The stretch failure ratios of APC3, PDC3, and PDL2 were the highest among the APC, PDC, and PDL groups; however, there were no significant differences between the other groups.

### 3.3 The influence of loading direction/test region/density on stiffness

**FIGURE 6:**
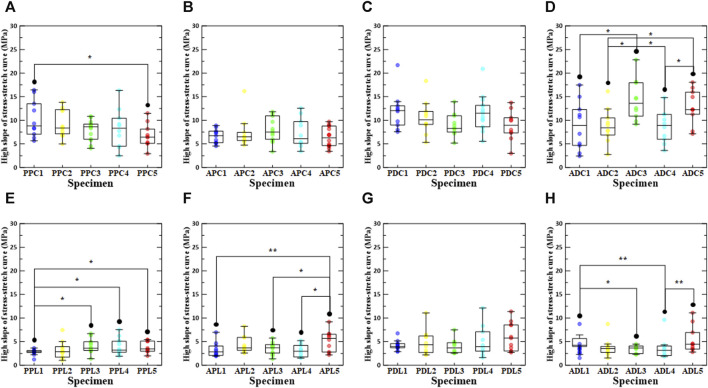
High slope of stress-stretch curve according to density increase ratio and test region. The first row shows circumferential specimens [**(A)** PPC, **(B)** APC, **(C)** PDC, and **(D)** ADC], and the second row shows longitudinal specimens [**(E)** PPL, **(F)** APL, **(G)** PDL, and **(H)** ADL].

Figure 6 shows the results of the high slope of the stress-stretch curve for all specimens in the comparative study. The results of the high slope in the loading direction confirmed that the high slope in the circumferential direction was significantly greater than that in the longitudinal direction. However, there was no statistically significant difference between APC5 and APL5. In the regional comparison of high slope under load in the circumferential direction, it was confirmed that PDC1, PDC2, ADC3, PDC4, and ADC5 (density increase rates of 0%, 0.1%–3%, 3%–6%, 6%–9%, and 9%–12%, respectively) had the highest high slope values. Moreover, in the regional comparison of high slope under load in the longitudinal direction, ADL1, PDL2, APL3, PDL4, and PDL5 (density increase rates of 0%, 0.1%–3%, 3%–6%, 6%–9%, and 9%–12% density increase rate) had the highest high slope values. In a comparative study of the high slope in relation to the increase in density, PPC1, PDC1, APC3, ADC3, PPL3, PDL5, APL5, and ADL5 were determined to be the highest. ADC was greater than APC, regardless of the density increase between the distal and proximal regions, and PDC was greater than PPC. In addition, a comparison between the proximal and anterior regions confirmed that the PPC appeared to be greater than the APC, regardless of the increase in density. With regards to ADL and PDL, in the group without stabilization treatment, ADL1 was greater than PDL1, whereas in the group with stabilization treatment, all PDL groups were greater than the ADL group.

### 3.4 Mechanical characteristic correlations

**FIGURE 7:**
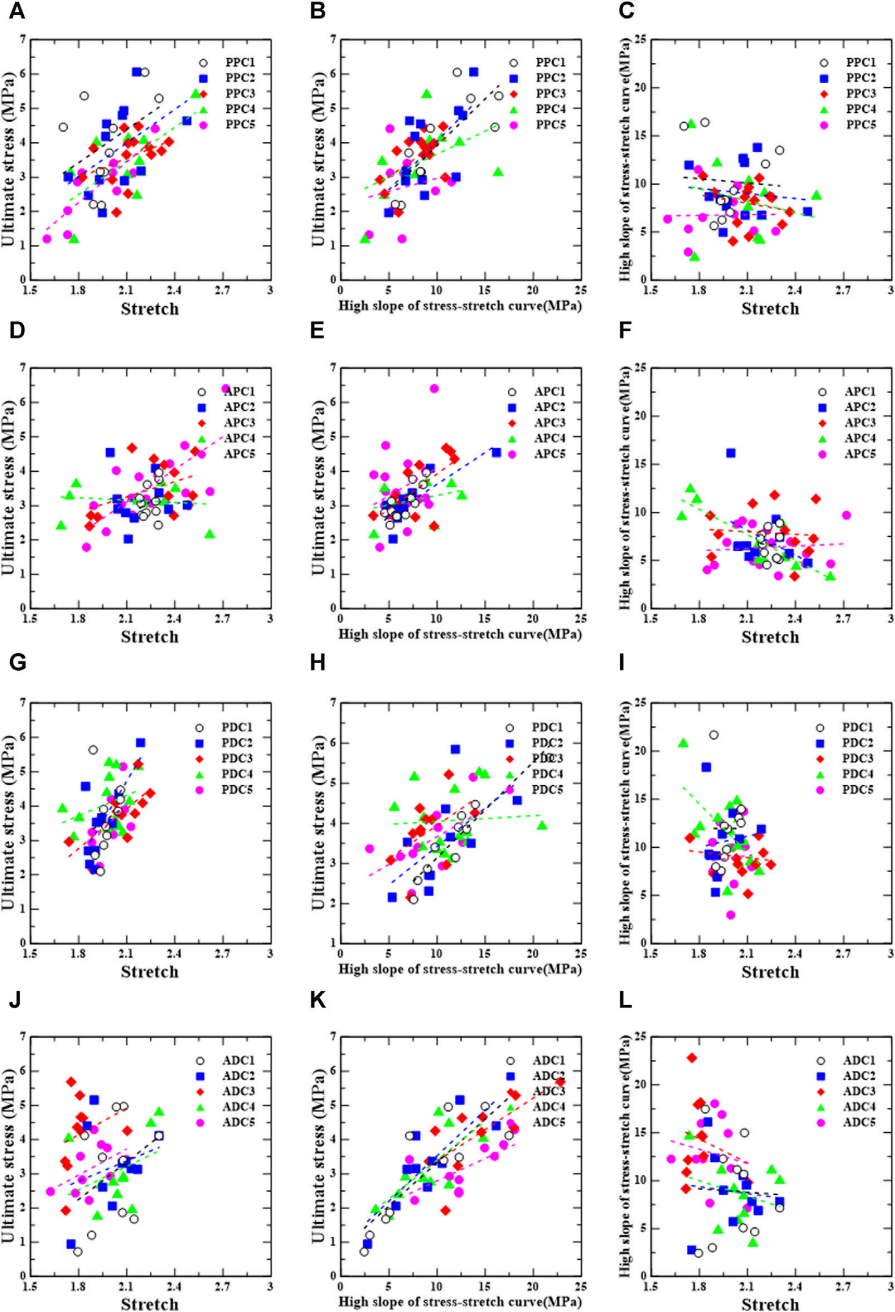
Circumferential group: correlation between ultimate stress, stretch, and relative high slope of stress-stretch curve according to test region [PPC **(A–C)**, APC **(D–F)**, PDC **(G–I)**, and ADC **(J–L)**].

**FIGURE 8:**
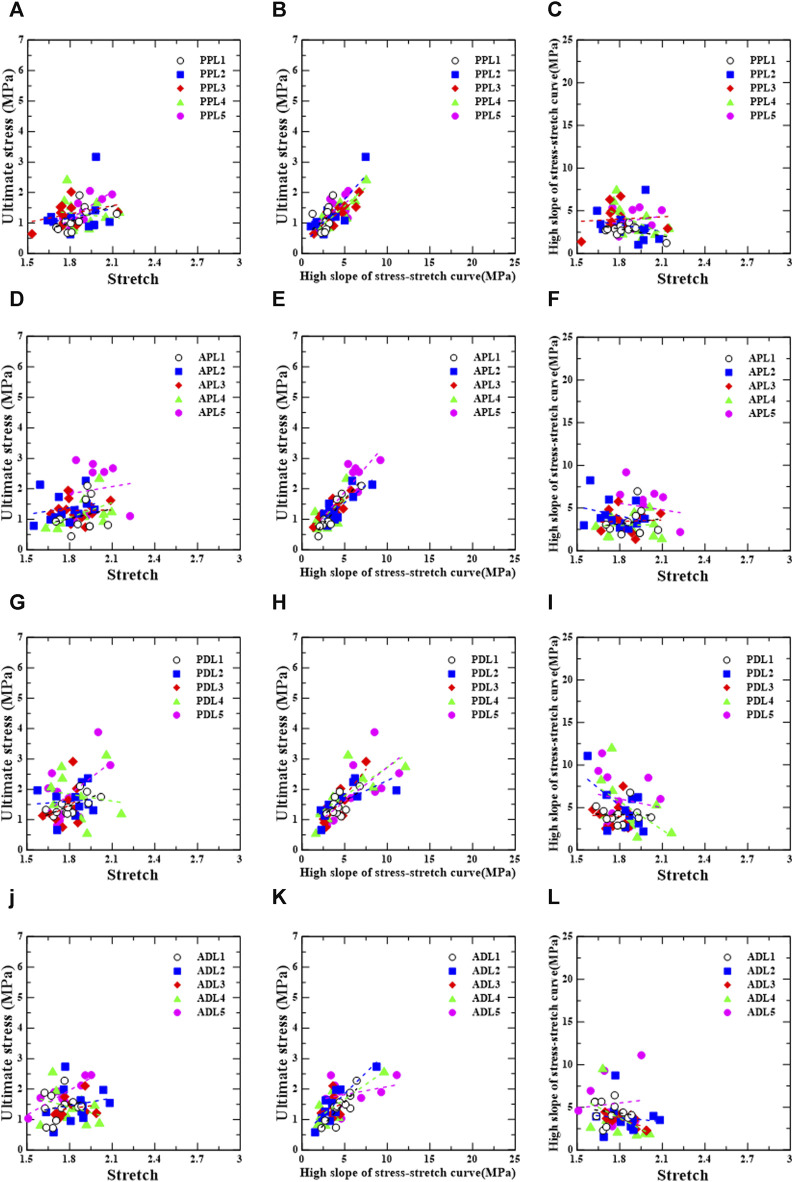
Longitudinal group: correlation between ultimate stress, stretch, and relative high slope of stress-stretch curve according to test region [PPL **(A–C)**, APL **(D–F)**, PDL **(G–I)**, and ADL **(J–L)**].

Figures 7, 8 show the linear regression plots for each region, loading direction, and density increase between the ultimate stress, stretch, and slope.

For the posterior/proximal/circumferential group, ultimate stress has a correlation, σ_u_ = 3.26λ_u_ − 3.37 (r = 0.670, *p* = 0.034), and a strong correlation, σ_u_ = 3.94λ_u_ − 4.84 (r = 0.857, *p* = 0.002), with stretch (λ_u_) for PPC4 and PPC5, respectively (Figure 7A).

Ultimate stress has a strong correlation, σ_u_ = 0.27E_u_ + 1.19 (r = 0.803, *p* = 0.005), and a correlation, σ_u_ = 0.28E_u_ + 1.22 (r = 0.653, *p* = 0.021), with the high slope (E_u_) for PPC1 and PPC2, respectively (Figure 7B).

For the anterior/proximal/circumferential group, ultimate stress correlates strongly with stretch, $\sigma_{u}=3.16\lambda_{u}-3.54$ (r = 0.742, *p* = 0.001), for APC5; However, in APC4, the high slope correlates strongly with stretch, $E_{h}=-8.80\lambda_{u}+26.2$ (r = −0.861, *p* = 0.001) (Figures 7D,F). Ultimate stress has a strong correlation, $\sigma_{u}=0.26E_{u}+1.38$ (r = 0.800, *p* = 0.003), and correlations, $\sigma_{u}=0.18E_{u}+1.78$ and $\sigma_{u}=0.20E_{u}+1.88$ (r = 0.685, *p* = 0.029; r = 0.658, *p* = 0.020), with the high slope for APC1, APC2, and APC3, respectively (Figure 7E).

For the posterior/distal/circumferential group, no correlations were found between the ultimate stress and stretch, ultimate stress and high slope, or high slope and stretch (Figures 7G–I).

For the anterior/distal/circumferential group, ultimate stress has strong correlations, $\sigma_{u}=0.25E_{u}+0.79$ ; $\sigma_{u}=0.27E_{u}+0.87$ , $\sigma_{u}=0.18E_{u}+1.53$ , and $\sigma_{u}=0.23E_{u}+1.13$ (r = 0.844, *p* = 0.002; r = 0.817, *p* = 0.004; r = 0.732, *p* = 0.016; r = 0.744, *p* = 0.014), and correlations, $\sigma_{u}=0.13E_{u}+1.49$ (r = 0.691, *p* = 0.027), with the high slope for ADC1, ADC2, ADC3, ADC4, and ADC5, respectively (Figure 7K).

For the posterior/proximal/longitudinal group, ultimate stress has strong correlations, $\sigma_{u}=0.31E_{u}+0.24$ , $\sigma_{u}=0.21E_{u}+0.44$ , and $\sigma_{u}=0.24E_{u}+0.33$ (r = 0.818, *p* = 0.002; r = 0.854, *p* = 0.0001; r = 0.865, *p* = 0.0001), with the high slope for PPL2, PPL3, and PPL4, respectively (Figure 8B). In PPL5, there is a strong correlation between ultimate stress and stretch, $\sigma_{u}=3.04\lambda_{u}+0.66$ (r = 0.754, *p* = 0.012) (Figure 8A).

For the anterior/proximal/longitudinal group, ultimate stress has a very strong correlation, $\sigma_{u}=0.32E_{u}+0.26$ (r = 0.951, *p* = 0.0001), a correlation, $\sigma_{u}=0.23E_{u}+0.44$ (r = 0.594, *p* = 0.042), strong correlations, $\sigma_{u}=0.23E_{u}+0.51$ and $\sigma_{u}=0.33E_{u}+0.26$ (r = 0.830, *p* = 0.001; r = 0.770, *p* = 0.009), with the high slope for APL1, APL2, APL3, and APL5, respectively (Figure 8E). In APL4, there is a correlation between ultimate stress and stretch, $\sigma_{u}=1.63\lambda_{u}-1.88$ (r = 0.682, *p* = 0.021) (Figure 8D).

For the posterior/distal/longitudinal group, ultimate stress has correlations, $\sigma_{u}=0.18E_{u}+0.72$ and $\sigma_{u}=0.13E_{u}+0.97$ (r = 0.585, *p* = 0.046; r = 0.697, *p* = 0.025), strong correlations, $\sigma_{u}=0.35E_{u}+0.0053$ , $\sigma_{u}=0.19E_{u}+0.85$ , and $\sigma_{u}=0.22E_{u}+0.48$ (r = 0.871, *p* = 0.001; r = 0.760, *p* = 0.011; r = 0.717, *p* = 0.013) with a high slope for PDL1, PDL2, PDL3, PDL4, and PDL5, respectively (Figure 8H). In PDL1, there is a correlation between ultimate stress and stretch, $\sigma_{u}=1.87\lambda_{u}-1.92$ (r = 0.673, *p* = 0.017) (Figure 8G).

For the anterior/distal/longitudinal group, ultimate stress has strong correlations, $\sigma_{u}=0.31E_{u}+0.053$ , $\sigma_{u}=0.28E_{u}+0.46$ , and $\sigma_{u}=0.20E_{u}+0.69$ (r = 0.844, *p* = 0.001; r = 0.782, *p* = 0.004; r = 0.806, *p* = 0.005), with the high slope for ADL1, ADL2, and ADL4, respectively (Figure 8K). In ADL3, the high slope is strongly correlated with stretch, $E_{u}=-5.18\lambda_{u}+12.9$ (r = −0.736, *p* = 0.010), whereas in ADL5, ultimate stress is strongly correlated with stretch, $\sigma_{u}=2.71\lambda_{u}-2.92$ (r = 0.857, *p* = 0.002) (Figures 8J, L).

## 4 Discussion

This study investigated the mechanical behavior of thoracic aortic tissue to assess the ultimate mechanical properties of biological tissues as a result of cross-link variations. Uniaxial tensile tests were conducted on porcine thoracic aorta specimens. The thoracic aorta was dissected into the anterior, posterior, proximal, and distal regions, and specimens were prepared for each region by considering the circumferential and longitudinal loading directions. In addition, the stabilization treatment induced variations in the cross-links in porcine aortic tissues; thus, the uniaxial tensile behavior in response to density variations was examined. From the stress-stretch curves, the ultimate stress, stretch at ultimate stress, and high slope were derived and analyzed to highlight the effects of specimen location, orientation, and density variation on the measured mechanical properties.

### 4.1 Anisotropic mechanical characteristics along the loading directions and test regions

In this study, circumferential and longitudinal uniaxial tensile tests were conducted to evaluate the mechanical properties of the aortic tissue under a uniaxial tensile load. Consequently, the ultimate stress and high slope values were significantly higher in the circumferential direction than in the longitudinal direction, and the stretch was higher in the circumferential direction than in the longitudinal direction, except for the results of the comparative analysis between ADC3 and ADL3. Pichamuthu et al. (2013), Duprey et al. (2010), Khanafer et al. (2011), Pham et al. (2013), Sokolis et al. (2012), Garcia-Herrera et al. (2012), Ferrara et al. (2016), and Xuan et al. (2021) confirmed that the ultimate stress and slope values were comparable. These results are consistent with previous findings (Sokolis et al., 2012; Tsamis et al., 2013) that collagen fibers are aligned more densely in the circumferential direction than in the longitudinal direction, regardless of stabilization treatment.

In the regional analysis for the median value of the normal aorta without the stabilization treatment, the ultimate stress of PPC1 was greater than that of PDC1, whereas the ultimate stress of APC1 was lower than that of ADC1. In addition, the ultimate stresses of PPL1 and APL1 were lower than those of PDL1 and ADL1, and those of PPC1 and PDC1 were higher than those of APC1 and ADC1. PPL1 had a higher ultimate stress than APL1, whereas PDL1 had a lower ultimate stress than APL1. These outcomes were comparable to those of our previous study, with the exception of the comparisons between PPC1, PDC1, PPL1, and APL1 (Ryu et al., 2022). Among the comparative analysis results for the high slope, the circumferential loading direction results were found to be quite similar to those of our previous study (PPC1 PDC1, APC1 ADC1, PPC1 APC1, and PDC1 ADC1), and the longitudinal loading direction results confirmed that PDL1 and ADL1 were higher than PPL1 and APL1, respectively, as in a previous study. However, the results indicated that PPL1 and ADL1 were greater than APL1 and PDL1, that differed from those of previous studies. Depending on the number of tensile tests, size of the test specimen, and test method, these differences may appear in the comparative analysis of certain areas (Pei et al., 2021).

### 4.2 Mechanical characteristics for the density variation

A previous study confirmed that collagen cross-links are elevated in aortic aneurysms and Marfan tissues. Hayashi and Hirayama (2017) and Wells et al. (1998) evaluated the change in the modulus of elasticity of the aortic wall due to collagen cross-linking by examining the mechanical behavior and degree of collagen cross-linking using a thermodynamic approach, such as hydrothermal isometric tension.

Based on these studies, we discovered density-dependent trends in the uniaxial tensile behavior of some groups of normal thoracic aorta tissue samples and stabilized thoracic aorta tissue samples. In the experimental results for the circumferential loading direction, PPC1 showed the highest ultimate stress among the PPC groups, whereas PPC5 showed the lowest ultimate stress. Although there was no tendency for the ultimate stress to increase gradually with increasing density, the PPC1 and PPC5 had significantly different ultimate stress values. In addition, statistical analysis confirmed that the ultimate stress of PPC5 differed significantly from those of PPC1, PPC2, and PPC3, with the exception of PPC4. This indicates that the increase in density in the PPC group influenced the ultimate stress value of the specimen at some point. In the experimental results of the APL and ADL groups under longitudinal loading, the ultimate stresses of APL5 and ADL5 were the highest and were significantly greater than those of APL1 and ADL1.

Results comparable to those of the ultimate stress analysis were observed in the density-dependent change in the high slope. In particular, the results of the comparative analysis of the high slopes of PPC1 and PPC5 differed significantly, and the high slopes of PPL1 and APL1 differed significantly from those of PPL3/PPL4/PPL5 and APL3/APL4/APL5. These findings imply that the increase in the density of the local aortic region as a result of cross-linking may affect the ultimate stress and maximum stiffness.

Additionally, the results of a complex comparative analysis based on density and region confirmed the existence of specific trends among the groups. In the comparative analysis of the ultimate stress between the PPC and PDC groups, the ultimate stress of the PPC group was greater than that of the PDC group. However, the ultimate stress of the PDC group was greater than that of the PPC group in environments with a density of at least 3%. In addition, in the comparative analyses between the ADC and APC groups, and the PPC and APC groups, the magnitude of the ultimate stress was reversed when the density was increased by at least 6% and 9%, respectively. In the high-slope comparison analysis between the ADL and PDL groups, stabilization treatment confirmed that the PDL group had a higher slope than the ADL group. Moreover, in the high slope comparison analysis between the APC and ADC, PPL and PDL, and PPC and APC groups, the high slope of the ADC, PDL, and PPC groups was greater than that of the APC, PPL, and APC groups, respectively. No discernible patterns were observed in a comparative analysis of the ultimate stress and high slope based on the density of the remaining groups. Increasing tissue density resulting from cross-linking in specific regions of an aortic aneurysm can influence its mechanical properties, as shown by these findings. Particularly, the extent to which mechanical properties vary in relation to the degree of cross-linking could assist in reducing some of the conflict concerning studies on anisotropy in the aortic wall (Iliopoulos et al., 2009a; Iliopoulos et al., 2009b; Choudhury et al., 2009; Duprey et al., 2010; Lindeman et al., 2010; Khanafer et al., 2011).

### 4.3 Correlation between mechanical characteristic and density increase ratio

This study investigated the correlation between the mechanical characteristics of aortic tissue and the rate of density increase (Supplementary Figures S5, 6). It was determined that there was no correlation between density variation and most regional and mechanical characteristics, whereas density increase ratio was significantly weakly negatively correlated with ultimate stress and high slope in the PPC group, $\sigma_{u}=-12.4E_{u}+16.5$ and $\sigma_{u}=-30.9E_{u}+40.8$ (r = −0.354, *p* = 0.008; r = −0.318, *p* = 0.017). In addition, it was determined that the high slope in the PPL group had a significant weak positive correlation with the density increase ratio, $\sigma_{u}=9.66E_{u}-6.53$ (r = 0.304, *p* = 0.022).

According to the increase in density illustrated in Sections 3.1 and 3.3, these results were evaluated more conservatively than the results of the comparative analysis of the ultimate stress and high slope. These differences could be attributed to differences in the comparative analysis techniques. The results in Section 3 were examined by dividing the density variation by section, whereas the correlation study of the density increase rate was examined individually without dividing the section; therefore, it was regarded as being conservatively evaluated. In addition, in the present study, given that uniaxial tensile tests were conducted on specimens whose density increased by only 12%, it is possible that no correlation was observed in the other groups.

### 4.4 Limitations

The main aim of this study was to increase our understanding of the density variation of the thoracic aorta tissue through cross-linking and present the results of a uniaxial tensile test based on this variation. However, this study had several limitations.

The fact that only uniaxial tensile tests were conducted to investigate the mechanical properties of thoracic aortic tissues is a major limitation. Biaxial tensile testing can significantly aid in the investigation of cross-link-induced anisotropic mechanical behavior. In addition, the biaxial tensile test is an essential method for assessing the wall properties of aortic aneurysm tissue in relation to cross-links, as the stiffness motion of collagen fibers is correlated with the non-affine motion of collagen and the rotational motion of fibers (Krasny et al., 2017; Krasny et al., 2018). On the other hand, uniaxial tensile test methods are advantageous for investigating local variations in the heterogeneous properties of blood vessels (Sacks and Sun, 2003; Holzapfel and Ogden, 2009). Therefore, in this study, uniaxial tensile tests were conducted to determine the most extreme values of the mechanical properties of the aortic tissue, and adjacent specimens were tested in different directions to obtain reliable information on the anisotropic response of the tissue. The current study indicates the first endeavor to examine the impact of cross-links on the aortic wall’s behavior. Further studies are planned to investigate the rotational motion of fibers and the non-affine characteristics of collagen motion as determined by biaxial tensile testing. The lack of investigation of tissue microstructure is an additional limitation of the present study. In other words, the microstructure of the aortic tissue used for cryopreservation has not been reviewed, and studies on the effects of cryopreservation on the mechanical properties of aortic tissue have produced contradictory findings (Venkatasubramanian et al., 2006; Chow and Zhang, 2011; Hemmasizadeh et al., 2012). In this study, we compared the mechanical behaviors of non-frozen and frozen aortic tissues and found that cryopreservation had no effect on the mechanical behavior of the aortic tissue. In addition, pigs aged 6 months were used in this study, which is associated considered the age at which aneurysms occur. Because the number of unstable cross-links is low in adult pigs, the range in which the density can be increased by the stabilization treatment may be limited. Additional studies are intended to be conducted in the future concerning cross-links formed in the aorta of juvenile pigs and fetuses. Trends in the effect of cross-links on mechanical properties will be assessed in association with the findings of this study. In order to predict abdominal aortic aneurysm rupture, we therefore intend to develop a finite element-based index that characterizes the behavior of aortic tissue in response to a broad range of cross-linking and density increase rates.

## 5 Conclusion

Using uniaxial tensile testing, we investigated the mechanical properties of the aortic wall in relation to the rate of density increase, the test region, and the loading direction. The current study focused on the mechanical characteristics of the aorta through the association between variations in cross-links and the rate of density increase. Notably, variations in aortic tissue tension and steep inclines were detected in specific areas as the density rose. Interestingly, variations in aortic tissue ultimate stress and high slopes were observed in specific areas as the density increased. In particular, the PPC group experienced greater ultimate stress than the PDC group; however, a reversal of this pattern was observed when the rate of density increase passed 3%. Additionally, when the density increase rates in the comparison study between the ADC and APC group and the PPC and APC group exceeded 6% and 9%, respectively, a reversal switched occur. A reversal phenomenon was observed in the high slope comparison between the PDL and ADL groups as a result of the stabilization treatment. Additional studies are necessary to determine the mechanisms governing variations in mechanical behavior across a wide range of density increases, as indicated by these results. Consequently, our plan is to conduct further cross-link-related experiments in the future in order to develop auxiliary indicators that can be utilized for predicting aortic rupture in patients diagnosed with aortic disease. The results of these investigations could potentially inform future recommendations regarding preventive treatment.

## Data Availability

The original contributions presented in the study are included in the article/Supplementary Material, further inquiries can be directed to the corresponding author.
