# Supplementary material for: Investigating the influence of collagen cross-linking on mechanical properties of thoracic aortic tissue
Source: Front Bioeng Biotechnol. 2024 Feb 27;12:1305128. doi: 10.3389/fbioe.2024.1305128 (PMC10928930; doi:10.3389/fbioe.2024.1305128)
Supplement: Supplementary file 1 [file DataSheet1.docx]

Supplementary Material

# Supplementary Figures and Tables

For more information on Supplementary Material and for details on the different file types accepted, please see [here](https://www.frontiersin.org/guidelines/author-guidelines#supplementary-material).

## Supplementary Figures


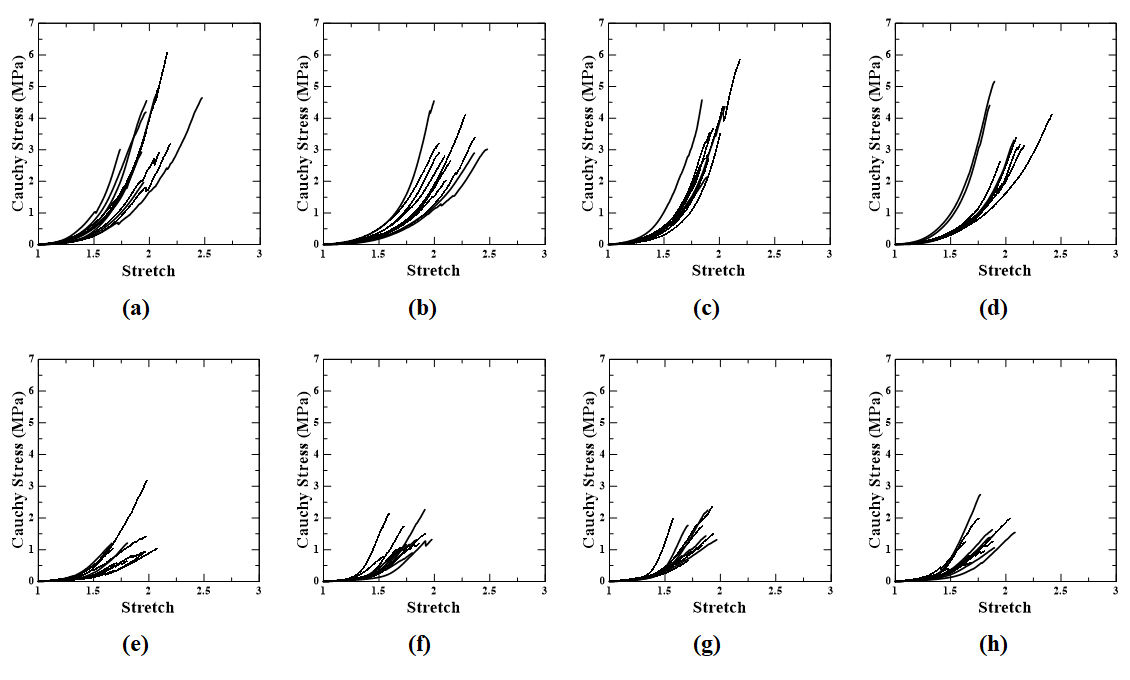


**Supplementary Figure 1.** Uniaxial stress-stretch curve of test regions varying in density from 0.1% to 3%. First row shows circumferential specimens ((a) PPC2, (b) APC2, (c) PDC2, (d) ADC2), and second row shows longitudinal specimens ((e) PPL2, (f) APL2, (g) PDL2, (h) ADL2).


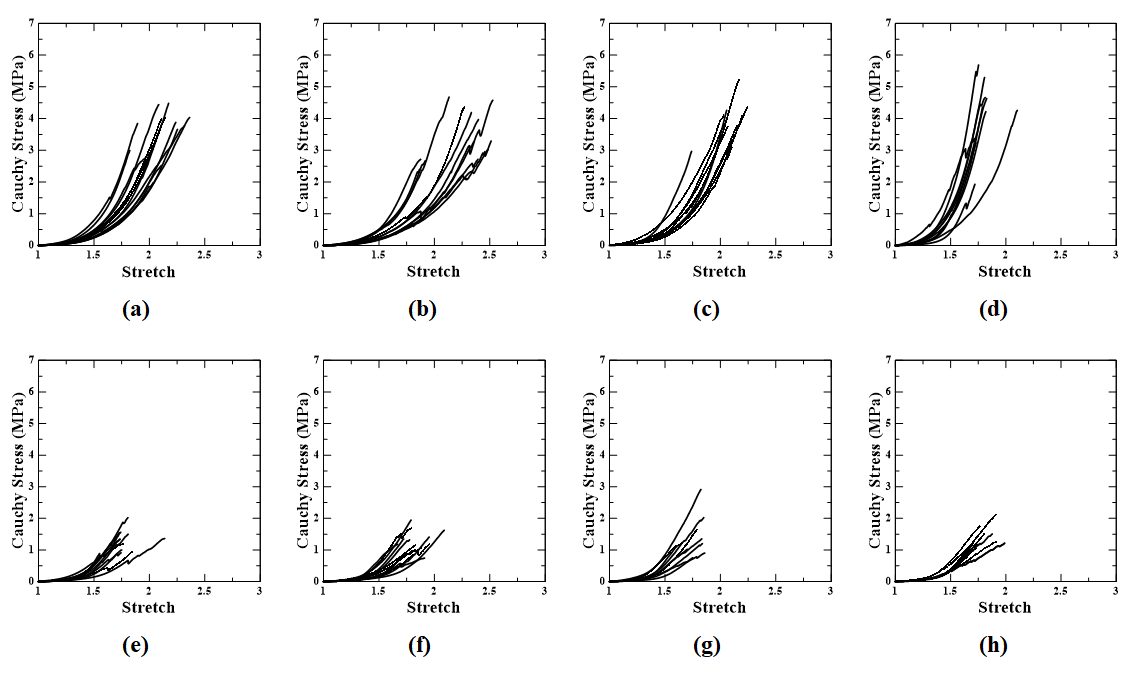


**Supplementary Figure 2.** Uniaxial stress-stretch curve of test regions varying in density from 3% to 6%. First row shows circumferential specimens ((a) PPC3, (b) APC3, (c) PDC3, (d) ADC3), and second row shows longitudinal specimens ((e) PPL3, (f) APL3, (g) PDL3, (h) ADL3).


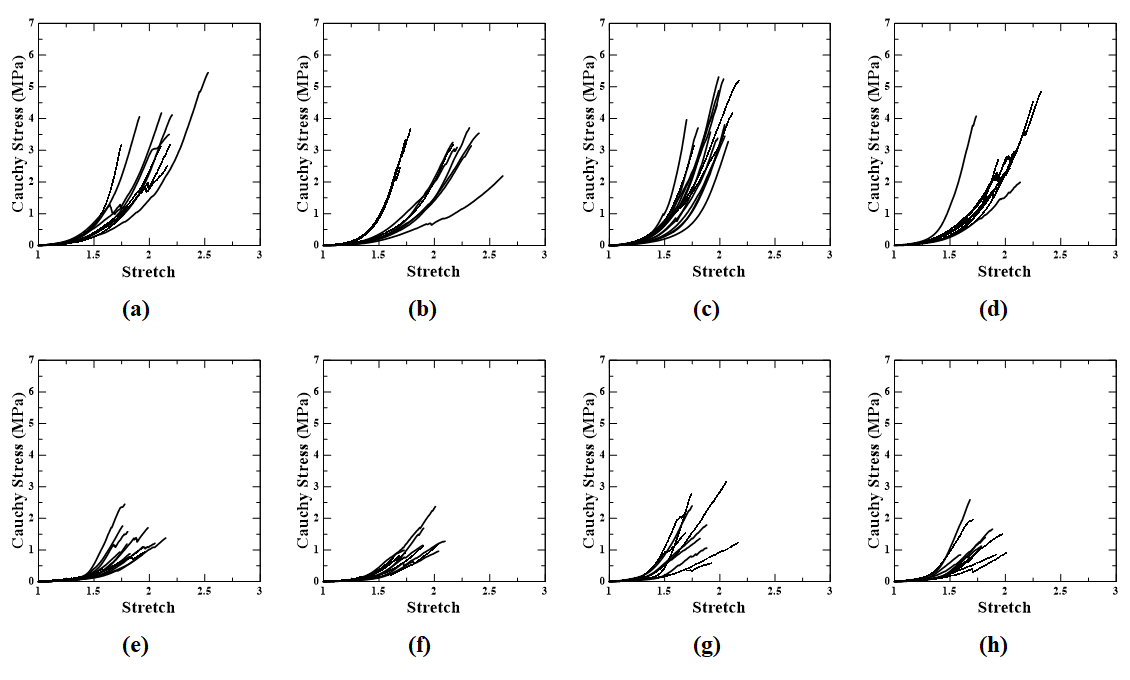


**Supplementary Figure 3.** Uniaxial stress-stretch curve of test regions varying in density from 6% to 9%. First row shows circumferential specimens ((a) PPC4, (b) APC4, (c) PDC4, (d) ADC4), and second row shows longitudinal specimens ((e) PPL4, (f) APL4, (g) PDL4, (h) ADL4).


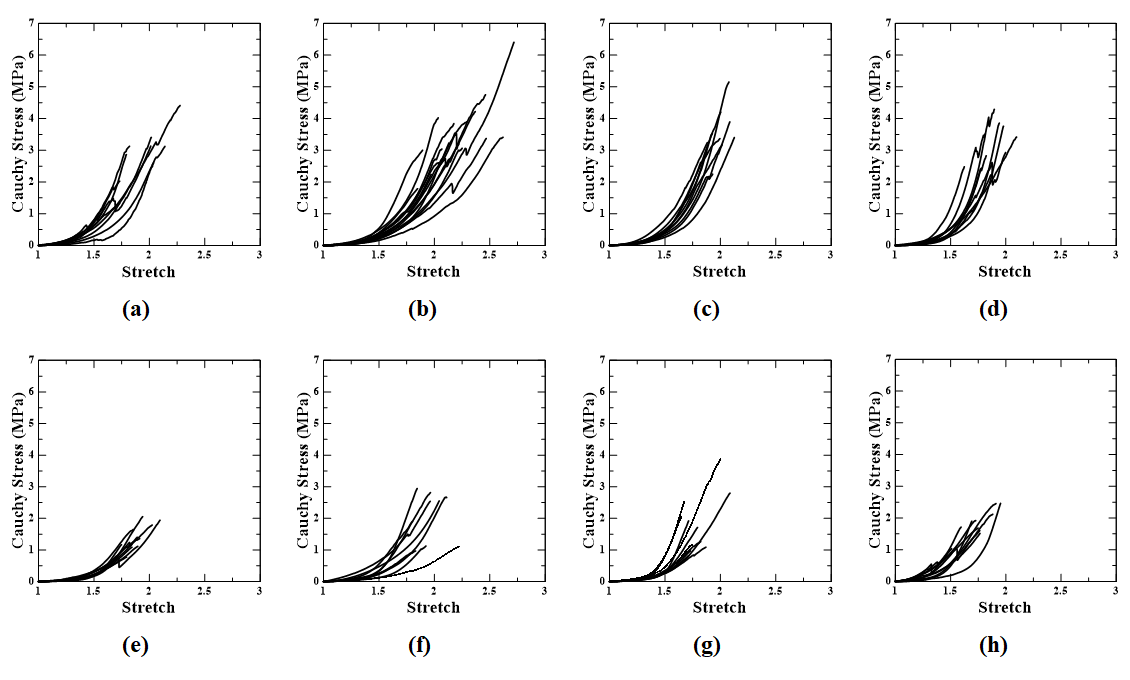


**Supplementary Figure 4.** Uniaxial stress-stretch curve of test regions varying in density from 9% to 12%. First row shows circumferential specimens ((a) PPC5, (b) APC5, (c) PDC5, (d) ADC5), and second row shows longitudinal specimens ((e) PPL5, (f) APL5, (g) PDL5, (h) ADL5).


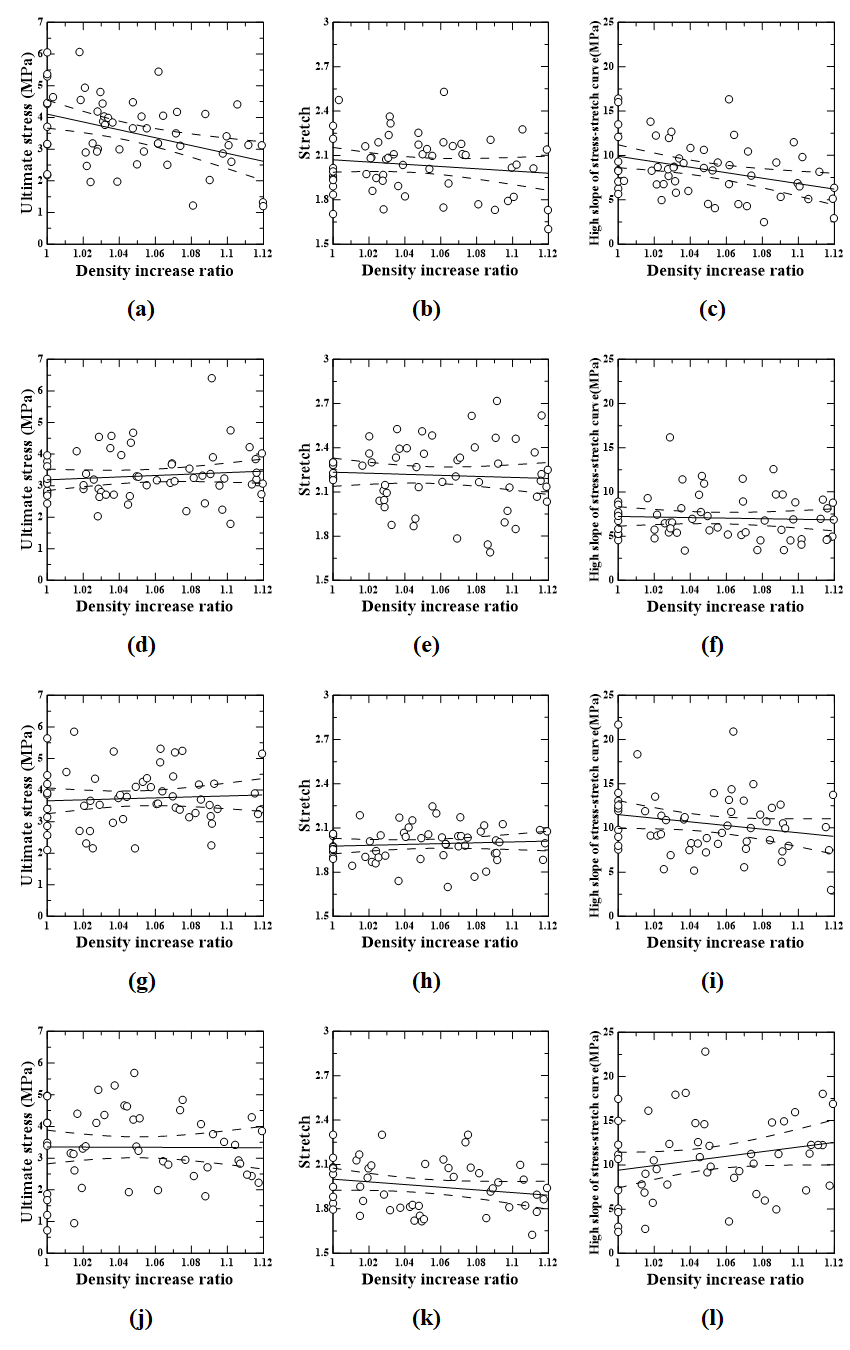


**Supplementary Figure 5.** Circumferential group: correlation between ultimate stress/stretch/high slope and density increase ratio according to test region (PPC (a–c), APC (d–f), PDC (g–i), ADC (j–l)).


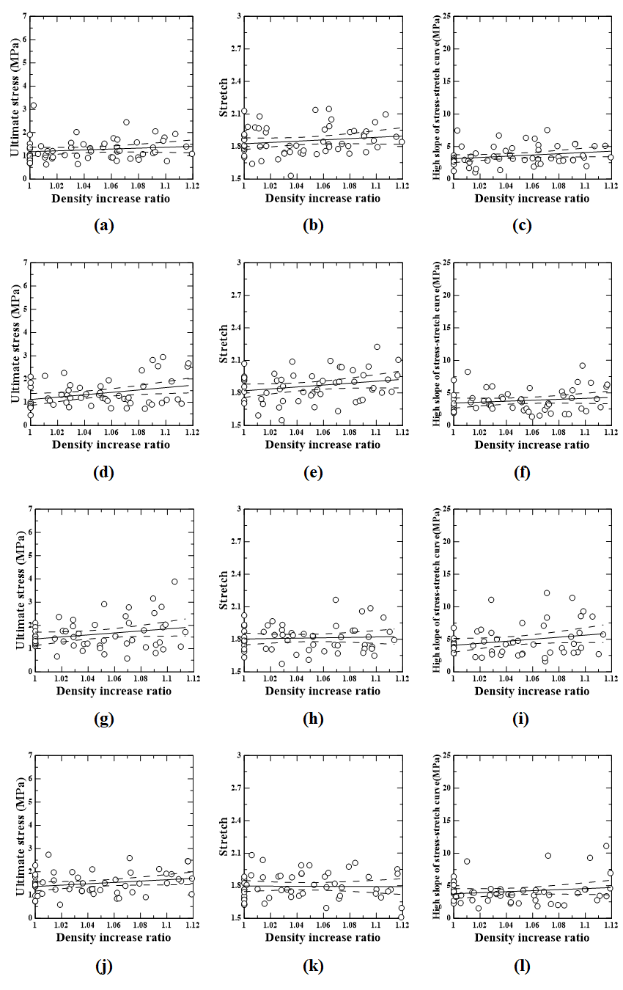


**Supplementary Figure 6.** Longitudinal group: correlation ultimate stress/stretch/high slope and density increase ratio according to test region (PPL (a–c), APL (d–f), PDL (g–i), ADL (j–l)).

## Supplementary Tables

**Supplementary Table 1.** Comparison of mechanical properties between circumferential and longitudinal loading directions.

| **Specimen of the posterior side of proximal descending thoracic aorta (PP)** | | | | | |
| --- | --- | --- | --- | --- | --- |
|  | Normal (PPC1/PPL1) | Density increase ratio of 0.1~3% (PPC2/PPL2) | Density increase ratio of 3~6% (PPC3/PPL3) | Density increase ratio of 6~9% (PPC4/PPL4) | Density increase ratio of 9~12% (PPC5/PPL5) |
| Ultimate stress  (MPa) | 4.00 (±0.424) vs 1.11 (±0.115) | 3.68 (2.92, 4.80) vs 1.04 (0.930, 1.21) | 3.58 (±0.193) vs 1.28 (±0.106) | 3.45 (±0.356) vs 1.28 (±0.133) | 2.72 (±0.309) vs 1.43 (±0.131) |
| p | <0.001 | <0.001 | <0.001 | <0.001 | 0.001 |
| Stretch | 1.98 (±0.055) vs 1.84 (±0.036) | 2.04 (±0.054) vs 1.86 (±0.046) | 2.11 (2.04, 2.24) vs 1.75 (1.74, 1.81) | 2.09 (±0.073) vs 1.89 (±0.035) | 1.92 (±0.067) vs 1.90 (±0.033) |
| p | 0.043 | 0.022 | <0.001 | 0.013 | 0.829 |
| High slope  (MPa) | 10.29 (±1.25) vs 2.76 (±0.185) | 9.12 (±0.818) vs 3.21 (±0.544) | 7.95 (±0.570) vs 4.00 (±0.434) | 8.30 (±1.31) vs 3.87 (±0.470) | 6.77 (±0.790) vs 3.91 (±0.388) |
| p | <0.001 | <0.001 | <0.001 | 0.008 | 0.004 |
|  | | | | | |
| **Specimen of the anterior side of proximal descending thoracic aorta (AP)** | | | | | |
|  | Normal (APC1/APL1) | Density increase ratio of 0.1~3% (APC2/APL2) | Density increase ratio of 3~6% (APC3/APL3) | Density increase ratio of 6~9% (APC4/APL4) | Density increase ratio of 9~12% (APC5/APL5) |
| Ultimate stress  (MPa) | 3.11 (±0.147) vs 1.11 (±0.173) | 3.15 (±0.228) vs 1.36 (±0.135) | 3.49 (±0.237) vs 1.29 (±0.100) | 3.21 (3.09, 3.54) vs 1.12 (0.901, 1.28) | 3.30 (3.04, 4.02) vs 2.22 (1.11, 2.67) |
| p | <0.001 | <0.001 | <0.001 | <0.001 | <0.001 |
| Stretch | 2.23 (2.19, 2.30) vs 1.92 (1.81, 1.94) | 2.18 (±0.051) vs 1.77 (±0.039) | 2.35 (2.13, 2.48) vs 1.82 (1.79, 1.95) | 2.14 (±0.098) vs 1.88 (±0.047) | 2.23 (±0.062) vs 1.95 (±0.044) |
| p | <0.001 | <0.001 | <0.001 | 0.023 | 0.004 |
| High slope  (MPa) | 6.72 (5.25, 7.70) vs 2.82 (2.08, 4.09) | 6.49 (5.73, 7.42) vs 3.66 (3.17, 5.88) | 7.86 (±0.764) vs 3.46 (±0.367) | 7.32 (±0.997) vs 2.99 (±0.383) | 6.37 (±0.515) vs 5.18 (±0.704) |
| p | <0.001 | 0.004 | <0.001 | 0.002 | 0.178 |
|  | | | | | |
| **Specimen of the posterior side of distal descending thoracic aorta (PD)** | | | | | |
|  | Normal (PDC1/PDL1) | Density increase ratio of 0.1~3% (PDC2/PDL2) | Density increase ratio of 3~6% (PDC3/PDL3) | Density increase ratio of 6~9% (PDC4/PDL4) | Density increase ratio of 9~12% (PDC5/PDL5) |
| Ultimate stress  (MPa) | 3.61 (±0.325) vs 1.46 (±0.091) | 3.53 (±0.364) vs 1.61 (±0.163) | 3.78 (±0.246) vs 1.42 (±0.203) | 4.07 (±0.196) vs 1.80 (±0.256) | 3.51 (±0.246) vs 1.83 (±0.283) |
| p | <0.001 | <0.001 | <0.001 | <0.001 | <0.001 |
| Stretch | 1.98 (±0.020) vs 1.81 (±0.032) | 1.95 (±0.034) vs 1.83 (±0.039) | 2.06 (±0.044) vs 1.77 (±0.028) | 1.98 (±0.034) vs 1.86 (±0.051) | 2.00 (1.93, 2.08) vs 1.75 (1.71, 1.87) |
| p | <0.001 | 0.030 | <0.001 | 0.054 | 0.004 |
| High slope  (MPa) | 11.97 (±1.29) vs 4.17 (±0.299) | 10.58 (±1.15) vs 4.87 (±0.861) | 9.00 (±0.708) vs 3.98 (±0.498) | 11.56 (±0.943) vs 5.06 (±1.04) | 8.89 (±1.00) vs 6.02 (±0.904) |
| p | <0.001 | <0.001 | <0.001 | <0.001 | 0.046 |
|  | | | | | |
| **Specimen of the anterior side of distal descending thoracic aorta (AD)** | | | | | |
|  | Normal (ADC1/ADL1) | Density increase ratio of 0.1~3% (ADC2/ADL2) | Density increase ratio of 3~6% (ADC3/ADL3) | Density increase ratio of 6~9% (ADC4/ADL4) | Density increase ratio of 9~12% (ADC5/ADL5) |
| Ultimate stress  (MPa) | 3.05 (±0.494) vs 1.42 (±0.131) | 3.22 (±0.378) vs 1.49 (±0.177) | 4.31 (3.37, 4.66) vs 1.21 (1.18, 1.51) | 3.10 (±0.327) vs 1.39 (±0.177) | 3.17 (±0.220) vs 1.84 (±0.137) |
| p | 0.009 | <0.001 | <0.001 | <0.001 | <0.001 |
| Stretch | 2.02 (±0.049) vs 1.73 (±0.026) | 2.02 (±0.052) vs 1.85 (±0.041) | 1.80 (1.73, 1.82) vs 1.81 (1.73, 1.91) | 2.05 (±0.052) vs 1.82 (±0.042) | 1.88 (±0.042) vs 1.75 (±0.043) |
| p | <0.001 | 0.015 | 0.725 | 0.003 | 0.050 |
| High slope  (MPa) | 8.89 (±1.64) vs 4.48 (±0.362) | 8.40 (6.88, 10.52) vs 3.52 (2.71, 3.99) | 14.29 (±1.36) vs 3.45 (±0.233) | 8.92 (5.98, 11.23) vs 3.15 (2.04, 4.08) | 12.26 (11.28, 15.97) vs 4.43 (3.44, 6.94) |
| p | 0.026 | 0.002 | <0.001 | 0.001 | <0.001 |

**Supplementary Table 2.** Comparison of mechanical properties for extraction locations in circumferential loading direction.

| **PPC vs APC** | | | | | |
| --- | --- | --- | --- | --- | --- |
|  | Normal (PPC1/APC1) | Density increase ratio of 0.1~3% (PPC2/APC2) | Density increase ratio of 3~6% (PPC3/APC3) | Density increase ratio of 6~9% (PPC4/APC4) | Density increase ratio of 9~12% (PPC5/APC5) |
| Ultimate stress  (MPa) | 4.00 (±0.424) vs 3.11 (±0.147) | 3.80 (±0.355) vs 3.15 (±0.228) | 3.58 (±0.193) vs 3.49 (±0.237) | 3.45 (±0.356) vs 3.15 (±0.156) | 2.72 (±0.309) vs 3.51 (±0.266) |
| p | 0.073 | 0.137 | 0.753 | 0.456 | 0.070 |
| Stretch | 1.95 (1.89, 2.02) vs 2.23 (2.19, 2.30) | 2.04 (±0.054) vs 2.18 (±0.051) | 2.11 (2.04, 2.24) vs 2.35 (2.13, 2.48) | 2.09 (±0.073) vs 2.14 (±0.098) | 1.92 (±0.067) vs 2.23 (±0.062) |
| p | 0.004 | 0.069 | 0.064 | 0.677 | 0.003 |
| High slope  (MPa) | 10.29 (±1.25) vs 6.60 (±0.449) | 8.36 (7.13, 12.25) vs 6.49 (5.73, 7.42) | 7.95 (±0.570) vs 7.86 (±0.764) | 8.30 (±1.31) vs 7.32 (±0.997) | 6.77 (±0.790) vs 6.37 (±0.515) |
| p | 0.018 | 0.048 | 0.930 | 0.556 | 0.659 |
|  | | | | | |
| **PPC vs PDC** | | | | | |
|  | Normal (PPC1/PDC1) | Density increase ratio of 0.1~3% (PPC2/PDC2) | Density increase ratio of 3~6% (PPC3/PDC3) | Density increase ratio of 6~9% (PPC4/PDC4) | Density increase ratio of 9~12% (PPC5/PDC5) |
| Ultimate stress  (MPa) | 4.00 (±0.424) vs 3.61 (±0.325) | 3.80 (±0.355) vs 3.53 (±0.364) | 3.58 (±0.193) vs 3.78 (±0.246) | 3.45 (±0.356) vs 4.07 (±0.196) | 2.72 (±0.309) vs 3.51 (±0.246) |
| p | 0.481 | 0.607 | 0.522 | 0.111 | 0.060 |
| Stretch | 1.98 (±0.055) vs 1.98 (±0.020) | 2.04 (±0.054) vs 1.95 (±0.034) | 2.12 (±0.040) vs 2.06 (±0.044) | 2.09 (±0.073) vs 1.98 (±0.034) | 1.92 (±0.067) vs 1.99 (±0.027) |
| p | 0.952 | 0.181 | 0.368 | 0.127 | 0.312 |
| High slope  (MPa) | 10.29 (±1.25) vs 11.97 (±1.29) | 9.12 (±0.818) vs 10.58 (±1.15) | 7.95 (±0.570) vs 9.00 (±0.708) | 8.30 (±1.31) vs 11.56 (±0.943) | 6.77 (±0.790) vs 8.89 (±1.00) |
| p | 0.362 | 0.299 | 0.251 | 0.049 | 0.114 |
|  | | | | | |
| **PPC vs ADC** | | | | | |
|  | Normal (PPC1/ADC1) | Density increase ratio of 0.1~3% (PPC2/ADC2) | Density increase ratio of 3~6% (PPC3/ADC3) | Density increase ratio of 6~9% (PPC4/ADC4) | Density increase ratio of 9~12% (PPC5/ADC5) |
| Ultimate stress  (MPa) | 4.00 (±0.424) vs 3.05 (±0.494) | 3.80 (±0.355) vs 3.22 (±0.378) | 3.58 (±0.193) vs 4.16 (±0.343) | 3.45 (±0.356) vs 3.10 (±0.327) | 2.72 (±0.309) vs 3.17 (±0.220) |
| p | 0.162 | 0.280 | 0.130 | 0.483 | 0.249 |
| Stretch | 1.98 (±0.055) vs 2.02 (±0.049) | 2.04 (±0.054) vs 2.02 (±0.052) | 2.11 (2.04, 2.24) vs 1.80 (1.73, 1.82) | 2.09 (±0.073) vs 2.05 (±0.052) | 1.92 (±0.067) vs 1.88 (±0.042) |
| p | 0.603 | 0.818 | <0.001 | 0.645 | 0.665 |
| High slope  (MPa) | 10.29 (±1.25) vs 8.89 (±1.64) | 9.12 (±0.818) vs 8.85 (±1.16) | 7.95 (±0.570) vs 14.29 (±1.36) | 8.30 (±1.31) vs 8.65 (±1.07) | 6.77 (±0.790) vs 12.87 (±1.16) |
| p | 0.506 | 0.848 | 0.001 | 0.837 | <0.001 |
|  | | | | | |
| **APC vs ADC** | | | | | |
|  | Normal (APC1/ADC1) | Density increase ratio of 0.1~3% (APC2/ADC2) | Density increase ratio of 3~6% (APC3/ADC3) | Density increase ratio of 6~9% (APC4/ADC4) | Density increase ratio of 9~12% (APC5/ADC5) |
| Ultimate stress  (MPa) | 3.11 (±0.147) vs 3.05 (±0.494) | 3.15 (±0.228) vs 3.22 (±0.378) | 3.49 (±0.237) vs 4.16 (±0.343) | 3.15 (±0.156) vs 3.10 (±0.327) | 3.51 (±0.266) vs 3.17 (±0.220) |
| p | 0.906 | 0.863 | 0.112 | 0.893 | 0.381 |
| Stretch | 2.23 (2.19, 2.30) vs 2.05 (1.88, 2.08) | 2.18 (±0.051) vs 2.02 (±0.052) | 2.35 (2.13, 2.48) vs 1.80 (1.73, 1.82) | 2.14 (±0.098) vs 2.05 (±0.052) | 2.23 (±0.062) vs 1.88 (±0.042) |
| p | 0.002 | 0.038 | <0.001 | 0.407 | <0.001 |
| High slope  (MPa) | 6.60 (±0.449) vs 8.89 (±1.64) | 6.49 (5.73, 7.42) vs 8.40 (6.88, 10.52) | 7.86 (±0.764) vs 14.29 (±1.36) | 7.32 (±0.997) vs 8.65 (±1.07) | 6.37 (±0.515) vs 12.87 (±1.16) |
| p | 0.207 | 0.151 | <0.001 | 0.374 | <0.001 |
|  | | | | | |
| **APC vs PDC** | | | | | |
|  | Normal (APC1/PDC1) | Density increase ratio of 0.1~3% (APC2/PDC2) | Density increase ratio of 3~6% (APC3/PDC3) | Density increase ratio of 6~9% (APC4/PDC4) | Density increase ratio of 9~12% (APC5/PDC5) |
| Ultimate stress  (MPa) | 3.11 (±0.147) vs 3.61 (±0.325) | 3.15 (±0.228) vs 3.53 (±0.364) | 3.49 (±0.237) vs 3.78 (±0.246) | 3.15 (±0.156) vs 4.07 (±0.196) | 3.51 (±0.266) vs 3.51 (±0.246) |
| p | 0.162 | 0.378 | 0.396 | 0.003 | 0.996 |
| Stretch | 2.23 (2.19, 2.30) vs 1.96 (1.94, 2.05) | 2.18 (±0.051) vs 1.95 (±0.034) | 2.35 (2.13, 2.48) vs 2.07 (2.03, 2.17) | 2.14 (±0.098) vs 1.98 (±0.034) | 2.23 (±0.062) vs 1.99 (±0.027) |
| p | <0.001 | 0.001 | 0.042 | 0.135 | 0.002 |
| High slope  (MPa) | 6.60 (±0.449) vs 11.97 (±1.29) | 6.49 (5.73, 7.42) vs 10.09 (9.13, 11.90) | 7.86 (±0.764) vs 9.00 (±0.708) | 7.32 (±0.997) vs 11.56 (±0.943) | 6.37 (±0.515) vs 8.89 (±1.00) |
| p | <0.001 | 0.034 | 0.289 | 0.006 | 0.021 |
|  | | | | | |
| **PDC vs ADC** | | | | | |
|  | Normal (PDC1/ADC1) | Density increase ratio of 0.1~3% (PDC2/ADC2) | Density increase ratio of 3~6% (PDC3/ADC3) | Density increase ratio of 6~9% (PDC4/ADC4) | Density increase ratio of 9~12% (PDC5/ADC5) |
| Ultimate stress  (MPa) | 3.61 (±0.325) vs 3.05 (±0.494) | 3.53 (±0.364) vs 3.22 (±0.378) | 3.78 (±0.246) vs 4.16 (±0.343) | 4.07 (±0.196) vs 3.10 (±0.327) | 3.51 (±0.246) vs 3.17 (±0.220) |
| p | 0.353 | 0.562 | 0.374 | 0.013 | 0.315 |
| Stretch | 1.98 (±0.020) vs 2.02 (±0.049) | 1.95 (±0.034) vs 2.02 (±0.052) | 2.07 (2.03, 2.17) vs 1.80 (1.73, 1.82) | 1.98 (±0.034) vs 2.05 (±0.052) | 1.99 (±0.027) vs 1.88 (±0.042) |
| p | 0.437 | 0.239 | 0.002 | 0.231 | 0.039 |
| High slope  (MPa) | 11.97 (±1.29) vs 8.89 (±1.64) | 10.58 (±1.15) vs 8.85 (±1.16) | 9.00 (±0.708) vs 14.29 (±1.36) | 11.56 (±0.943) vs 8.65 (±1.07) | 8.89 (±1.00) vs 12.87 (±1.16) |
| p | 0.157 | 0.302 | 0.002 | 0.057 | 0.018 |

**Supplementary Table 3.** Comparison of mechanical properties for extraction locations in longitudinal loading direction.

| **PPL vs APL** | | | | | |
| --- | --- | --- | --- | --- | --- |
|  | Normal (PPL1/APL1) | Density increase ratio of 0.1~3% (PPL2/APL2) | Density increase ratio of 3~6% (PPL3/APL3) | Density increase ratio of 6~9% (PPL4/APL4) | Density increase ratio of 9~12% (PPL5/APL5) |
| Ultimate stress  (MPa) | 1.11 (±0.115) vs 1.11 (±0.173) | 1.04 (0.930, 1.21) vs 1.24 (1.07, 1.73) | 1.28 (±0.106) vs 1.29 (±0.100) | 1.19 (0.931, 1.58) vs 1.12 (0.901, 1.28) | 1.38 (1.11, 1.79) vs 2.22 (1.11, 2.67) |
| p | 0.983 | 0.242 | 0.929 | 0.582 | 0.257 |
| Stretch | 1.84 (±0.036) vs 1.88 (±0.035) | 1.86 (±0.046) vs 1.77 (±0.039) | 1.75 (1.74, 1.81) vs 1.82 (1.79, 1.95) | 1.89 (±0.035) vs 1.88 (±0.047) | 1.90 (±0.033) vs 1.95 (±0.044) |
| p | 0.390 | 0.147 | 0.094 | 0.918 | 0.360 |
| High slope  (MPa) | 2.85 (2.69, 3.12) vs 2.82 (2.08, 4.09) | 2.85 (1.70, 3.95) vs 3.66 (3.17, 5.88) | 4.00 (±0.434) vs 3.46 (±0.367) | 3.87 (±0.470) vs 2.99 (±0.383) | 3.91 (±0.388) vs 5.18 (±0.704) |
| p | 0.833 | 0.097 | 0.353 | 0.167 | 0.132 |
|  | | | | | |
| **PPL vs PDL** | | | | | |
|  | Normal (PPL1/PDL1) | Density increase ratio of 0.1~3% (PPL2/PDL2) | Density increase ratio of 3~6% (PPL3/PDL3) | Density increase ratio of 6~9% (PPL4/PDL4) | Density increase ratio of 9~12% (PPL5/PDL5) |
| Ultimate stress  (MPa) | 1.11 (±0.115) vs 1.46 (±0.091) | 1.04 (0.930, 1.21) vs 1.62 (1.31, 1.97) | 1.28 (±0.106) vs 1.42 (±0.203) | 1.28 (±0.133) vs 1.80 (±0.256) | 1.43 (±0.131) vs 1.83 (±0.283) |
| p | 0.023 | 0.024 | 0.533 | 0.071 | 0.228 |
| Stretch | 1.84 (±0.036) vs 1.81 (±0.032) | 1.86 (±0.046) vs 1.83 (±0.039) | 1.75 (1.74, 1.81) vs 1.81 (1.70, 1.84) | 1.89 (±0.035) vs 1.86 (±0.051) | 1.90 (±0.033) vs 1.80 (±0.041) |
| p | 0.504 | 0.547 | 0.510 | 0.626 | 0.078 |
| High slope  (MPa) | 2.76 (±0.185) vs 4.17 (±0.299) | 3.21 (±0.544) vs 4.87 (±0.861) | 4.00 (±0.434) vs 3.98 (±0.498) | 3.87 (±0.470) vs 5.06 (±1.04) | 3.91 (±0.388) vs 6.02 (±0.904) |
| p | <0.001 | 0.112 | 0.972 | 0.273 | 0.051 |
|  | | | | | |
| **PPL vs ADL** | | | | | |
|  | Normal (PPL1/ADL1) | Density increase ratio of 0.1~3% (PPL2/ADL2) | Density increase ratio of 3~6% (PPL3/ADL3) | Density increase ratio of 6~9% (PPL4/ADL4) | Density increase ratio of 9~12% (PPL5/ADL5) |
| Ultimate stress  (MPa) | 1.11 (±0.115) vs 1.42 (±0.131) | 1.04 (0.930, 1.21) vs 1.46 (1.06, 1.97) | 1.34 (1.00, 1.53) vs 1.21 (1.18, 1.51) | 1.28 (±0.133) vs 1.39 (±0.177) | 1.43 (±0.131) vs 1.84 (±0.137) |
| p | 0.093 | 0.071 | 0.667 | 0.620 | 0.047 |
| Stretch | 1.84 (±0.036) vs 1.73 (±0.026) | 1.86 (±0.046) vs 1.85 (±0.041) | 1.75 (1.74, 1.81) vs 1.81 (1.73, 1.91) | 1.89 (±0.035) vs 1.82 (±0.042) | 1.90 (±0.033) vs 1.75 (±0.043) |
| p | 0.021 | 0.807 | 0.356 | 0.203 | 0.016 |
| High slope  (MPa) | 2.76 (±0.185) vs 4.48 (±0.362) | 2.85 (1.70, 3.95) vs 3.52 (2.71, 3.99) | 4.00 (±0.434) vs 3.45 (±0.233) | 3.19 (2.77, 5.07) vs 3.15 (2.04, 4.08) | 3.48 (2.97, 5.11) vs 4.43 (3.44, 6.94) |
| p | <0.001 | 0.450 | 0.289 | 0.385 | 0.257 |
|  | | | | | |
| **APL vs ADL** | | | | | |
|  | Normal (APL1/ADL1) | Density increase ratio of 0.1~3% (APL2/ADL2) | Density increase ratio of 3~6% (APL3/ADL3) | Density increase ratio of 6~9% (APL4/ADL4) | Density increase ratio of 9~12% (APL5/ADL5) |
| Ultimate stress  (MPa) | 1.11 (±0.173) vs 1.42 (±0.131) | 1.36 (±0.135) vs 1.49 (±0.177) | 1.25 (1.15, 1.62) vs 1.21 (1.18, 1.51) | 1.12 (0.901, 1.28) vs 1.26 (0.909, 1.66) | 2.22 (1.11, 2.67) vs 1.81 (1.60, 2.12) |
| p | 0.168 | 0.559 | 0.580 | 0.439 | 0.597 |
| Stretch | 1.88 (±0.035) vs 1.73 (±0.026) | 1.77 (±0.039) vs 1.85 (±0.041) | 1.85 (±0.033) vs 1.83 (±0.033) | 1.88 (±0.047) vs 1.82 (±0.042) | 1.95 (±0.044) vs 1.75 (±0.043) |
| p | 0.002 | 0.201 | 0.688 | 0.321 | 0.005 |
| High slope  (MPa) | 2.82 (2.08, 4.09) vs 4.25 (3.97, 5.66) | 3.66 (3.17, 5.88) vs 3.52 (2.71, 3.99) | 3.46 (±0.367) vs 3.45 (±0.233) | 2.96 (1.74, 4.20) vs 3.15 (2.04, 4.08) | 5.69 (2.81, 6.57) vs 4.43 (3.44, 6.94) |
| p | 0.048 | 0.538 | 0.982 | 0.481 | 0.880 |
|  | | | | | |
| **APL vs PDL** | | | | | |
|  | Normal (APL1/PDL1) | Density increase ratio of 0.1~3% (APL2/PDL2) | Density increase ratio of 3~6% (APL3/PDL3) | Density increase ratio of 6~9% (APL4/PDL4) | Density increase ratio of 9~12% (APL5/PDL5) |
| Ultimate stress  (MPa) | 1.11 (±0.173) vs 1.46 (±0.091) | 1.36 (±0.135) vs 1.61 (±0.163) | 1.29 (±0.100) vs 1.42 (±0.203) | 1.12 (0.901, 1.28) vs 1.66 (1.22, 2.39) | 2.22 (1.11, 2.67) vs 1.71 (1.09, 2.53) |
| p | 0.074 | 0.249 | 0.566 | 0.041 | 0.673 |
| Stretch | 1.88 (±0.035) vs 1.81 (±0.032) | 1.77 (±0.039) vs 1.83 (±0.039) | 1.85 (±0.033) vs 1.77 (±0.028) | 1.88 (±0.047) vs 1.86 (±0.051) | 1.95 (±0.044) vs 1.80 (±0.041) |
| p | 0.124 | 0.357 | 0.097 | 0.740 | 0.022 |
| High slope  (MPa) | 2.82 (2.08, 4.09) vs 3.96 (3.72, 4.58) | 3.66 (3.17, 5.88) vs 4.35 (2.68, 6.21) | 3.46 (±0.367) vs 3.98 (±0.498) | 2.99 (±0.383) vs 5.06 (±1.04) | 5.18 (±0.704) vs 6.02 (±0.904) |
| p | 0.075 | 0.742 | 0.405 | 0.087 | 0.478 |
|  | | | | | |
| **PDL vs ADL** | | | | | |
|  | Normal (PDL1/ADL1) | Density increase ratio of 0.1~3% (PDL2/ADL2) | Density increase ratio of 3~6% (PDL3/ADL3) | Density increase ratio of 6~9% (PDL4/ADL4) | Density increase ratio of 9~12% (PDL5/ADL5) |
| Ultimate stress  (MPa) | 1.46 (±0.091) vs 1.42 (±0.131) | 1.61 (±0.163) vs 1.49 (±0.177) | 1.21 (1.02, 1.64) vs 1.21 (1.18, 1.51) | 1.80 (±0.256) vs 1.39 (±0.177) | 1.83 (±0.283) vs 1.84 (±0.137) |
| p | 0.776 | 0.631 | 0.573 | 0.211 | 0.988 |
| Stretch | 1.81 (±0.032) vs 1.73 (±0.026) | 1.83 (±0.039) vs 1.85 (±0.041) | 1.77 (±0.028) vs 1.83 (±0.033) | 1.86 (±0.051) vs 1.82 (±0.042) | 1.80 (±0.041) vs 1.75 (±0.043) |
| p | 0.081 | 0.702 | 0.196 | 0.532 | 0.457 |
| High slope  (MPa) | 4.17 (±0.299) vs 4.48 (±0.362) | 4.35 (2.68, 6.21) vs 3.52 (2.71, 3.99) | 3.98 (±0.498) vs 3.45 (±0.233) | 3.93 (2.98, 7.09) vs 3.15 (2.04, 4.08) | 5.74 (3.02, 8.56) vs 4.43 (3.44, 6.94) |
| p | 0.520 | 0.360 | 0.356 | 0.257 | 0.833 |

**Supplementary Table 4.** Comparison of mechanical properties for density increase ratio in circumferential loading direction.

| **Specimen of the posterior side of proximal descending thoracic aorta (PP)** | | | | | |
| --- | --- | --- | --- | --- | --- |
|  | PPC1/PPC2 | PPC1/PPC3 | PPC1/PPC4 | PPC1/PPC5 | PPC2/PPC3 |
| Ultimate stress  (MPa) | 4.00 (±0.424) vs 3.80 (±0.355) | 4.00 (±0.424) vs 3.58 (±0.193) | 4.00 (±0.424) vs 3.45 (±0.356) | 4.00 (±0.424) vs 2.72 (±0.309) | 3.80 (±0.355) vs 3.58 (±0.193) |
| p | 0.723 | 0.389 | 0.332 | 0.025 | 0.596 |
| Stretch | 1.98 (±0.055) vs 2.04 (±0.054) | 1.98 (±0.055) vs 2.12 (±0.040) | 1.98 (±0.055) vs 2.09 (±0.073) | 1.98 (±0.055) vs 1.92 (±0.067) | 2.04 (±0.054) vs 2.12 (±0.040) |
| p | 0.437 | 0.047 | 0.236 | 0.478 | 0.253 |
| High slope  (MPa) | 10.29 (±1.25) vs 9.12 (±0.818) | 10.29 (±1.25) vs 7.95 (±0.570) | 10.29 (±1.25) vs 8.30 (±1.31) | 10.29 (±1.25) vs 6.77 (±0.790) | 9.12 (±0.818) vs 7.95 (±0.570) |
| p | 0.427 | 0.112 | 0.285 | 0.028 | 0.241 |
|  | | | | | |
|  | PPC2/PPC4 | PPC2/PPC5 | PPC3/PPC4 | PPC3/PPC5 | PPC4/PPC5 |
| Ultimate stress  (MPa) | 3.80 (±0.355) vs 3.45 (±0.356) | 3.80 (±0.355) vs 2.72 (±0.309) | 3.58 (±0.193) vs 3.45 (±0.356) | 3.58 (±0.193) vs 2.72 (±0.309) | 3.45 (±0.356) vs 2.72 (±0.309) |
| p | 0.491 | 0.036 | 0.719 | 0.021 | 0.141 |
| Stretch | 2.04 (±0.054) vs 2.09 (±0.073) | 2.04 (±0.054) vs 1.92 (±0.067) | 2.12 (±0.040) vs 2.09 (±0.073) | 2.12 (±0.040) vs 1.92 (±0.067) | 2.09 (±0.073) vs 1.92 (±0.067) |
| p | 0.577 | 0.161 | 0.732 | 0.012 | 0.096 |
| High slope  (MPa) | 9.12 (±0.818) vs 8.30 (±1.31) | 9.12 (±0.818) vs 6.77 (±0.790) | 7.95 (±0.570) vs 8.30 (±1.31) | 7.95 (±0.570) vs 6.77 (±0.790) | 8.30 (±1.31) vs 6.77 (±0.790) |
| p | 0.589 | 0.055 | 0.785 | 0.227 | 0.329 |
|  | | | | | |
| **Specimen of the anterior side of proximal descending thoracic aorta (AP)** | | | | | |
|  | APC1/APC2 | APC1/APC3 | APC1/APC4 | APC1/APC5 | APC2/APC3 |
| Ultimate stress  (MPa) | 3.11 (±0.147) vs 3.15 (±0.228) | 3.11 (±0.147) vs 3.49 (±0.237) | 3.11 (±0.147) vs 3.15 (±0.156) | 3.11 (±0.147) vs 3.51 (±0.266) | 3.15 (±0.228) vs 3.49 (±0.237) |
| p | 0.895 | 0.195 | 0.861 | 0.258 | 0.319 |
| Stretch | 2.23 (2.19, 2.30) vs 2.13 (2.05, 2.30) | 2.23 (2.19, 2.30) vs 2.35 (2.13, 2.48) | 2.23 (2.19, 2.30) vs 2.19 (1.78, 2.33) | 2.23 (2.19, 2.30) vs 2.20 (2.07, 2.46) | 2.13 (2.05, 2.30) vs 2.35 (2.13, 2.48) |
| p | 0.245 | 0.295 | 0.622 | 0.459 | 0.323 |
| High slope  (MPa) | 6.72 (5.25, 7.70) vs 6.49 (5.73, 7.42) | 6.60 (±0.449) vs 7.86 (±0.764) | 6.60 (±0.449) vs 7.32 (±0.997) | 6.60 (±0.449) vs 6.37 (±0.515) | 6.49 (5.73, 7.42) vs 7.50 (5.98, 10.94) |
| p | 0.833 | 0.178 | 0.524 | 0.751 | 0.391 |
|  | | | | | |
|  | APC2/APC4 | APC2/APC5 | APC3/APC4 | APC3/APC5 | APC4/APC5 |
| Ultimate stress  (MPa) | 3.15 (±0.228) vs 3.15 (±0.156) | 3.15 (±0.228) vs 3.51 (±0.266) | 3.49 (±0.237) vs 3.15 (±0.156) | 3.49 (±0.237) vs 3.51 (±0.266) | 3.15 (±0.156) vs 3.51 (±0.266) |
| p | 0.992 | 0.351 | 0.250 | 0.949 | 0.326 |
| Stretch | 2.18 (±0.051) vs 2.14 (±0.098) | 2.18 (±0.051) vs 2.23 (±0.062) | 2.35 (2.13, 2.48) vs 2.19 (1.78, 2.33) | 2.35 (2.13, 2.48) vs 2.20 (2.07, 2.46) | 2.14 (±0.098) vs 2.23 (±0.062) |
| p | 0.704 | 0.629 | 0.262 | 0.577 | 0.443 |
| High slope  (MPa) | 6.49 (5.73, 7.42) vs 6.10 (5.13, 9.72) | 6.49 (5.73, 7.42) vs 6.29 (4.65, 8.79) | 7.86 (±0.764) vs 7.32 (±0.997) | 7.86 (±0.764) vs 6.37 (±0.515) | 7.32 (±0.997) vs 6.37 (±0.515) |
| p | 0.762 | 0.429 | 0.663 | 0.104 | 0.360 |
|  | | | | | |
| **Specimen of the posterior side of distal descending thoracic aorta (PD)** | | | | | |
|  | PDC1/PDC2 | PDC1/PDC3 | PDC1/PDC4 | PDC1/PDC5 | PDC2/PDC3 |
| Ultimate stress  (MPa) | 3.61 (±0.325) vs 3.53 (±0.364) | 3.61 (±0.325) vs 3.78 (±0.246) | 3.61 (±0.325) vs 4.07 (±0.196) | 3.61 (±0.325) vs 3.51 (±0.246) | 3.53 (±0.364) vs 3.78 (±0.246) |
| p | 0.872 | 0.679 | 0.214 | 0.808 | 0.571 |
| Stretch | 1.98 (±0.020) vs 1.95 (±0.034) | 1.98 (±0.020) vs 2.06 (±0.044) | 1.98 (±0.020) vs 1.98 (±0.034) | 1.98 (±0.020) vs 1.99 (±0.027) | 1.95 (±0.034) vs 2.06 (±0.044) |
| p | 0.486 | 0.092 | 0.980 | 0.609 | 0.053 |
| High slope  (MPa) | 11.97 (±1.29) vs 10.58 (±1.15) | 11.97 (±1.29) vs 9.00 (±0.708) | 11.97 (±1.29) vs 11.56 (±0.943) | 11.97 (±1.29) vs 8.89 (±1.00) | 10.58 (±1.15) vs 9.00 (±0.708) |
| p | 0.432 | 0.052 | 0.794 | 0.075 | 0.245 |
|  | | | | | |
|  | PDC2/PDC4 | PDC2/PDC5 | PDC3/PDC4 | PDC3/PDC5 | PDC4/PDC5 |
| Ultimate stress  (MPa) | 3.53 (±0.364) vs 4.07 (±0.196) | 3.53 (±0.364) vs 3.51 (±0.246) | 3.78 (±0.246) vs 4.07 (±0.196) | 3.78 (±0.246) vs 3.51 (±0.246) | 4.07 (±0.196) vs 3.51 (±0.246) |
| p | 0.173 | 0.962 | 0.367 | 0.448 | 0.089 |
| Stretch | 1.95 (±0.034) vs 1.98 (±0.034) | 1.95 (±0.034) vs 1.99 (±0.027) | 2.06 (±0.044) vs 1.98 (±0.034) | 2.06 (±0.044) vs 1.99 (±0.027) | 1.98 (±0.034) vs 1.99 (±0.027) |
| p | 0.567 | 0.310 | 0.123 | 0.197 | 0.732 |
| High slope  (MPa) | 10.58 (±1.15) vs 11.56 (±0.943) | 10.58 (±1.15) vs 8.89 (±1.00) | 9.00 (±0.708) vs 11.56 (±0.943) | 9.00 (±0.708) vs 8.89 (±1.00) | 11.56 (±0.943) vs 8.89 (±1.00) |
| p | 0.519 | 0.280 | 0.054 | 0.926 | 0.072 |
|  | | | | | |
| **Specimen of the anterior side of distal descending thoracic aorta (AD)** | | | | | |
|  | ADC1/ADC2 | ADC1/ADC3 | ADC1/ADC4 | ADC1/ADC5 | ADC2/ADC3 |
| Ultimate stress  (MPa) | 3.05 (±0.494) vs 3.22 (±0.378) | 3.05 (±0.494) vs 4.16 (±0.343) | 3.05 (±0.494) vs 3.10 (±0.327) | 3.05 (±0.494) vs 3.17 (±0.220) | 3.22 (±0.378) vs 4.16 (±0.343) |
| p | 0.782 | 0.081 | 0.933 | 0.825 | 0.083 |
| Stretch | 2.02 (±0.049) vs 2.02 (±0.052) | 2.05 (1.88, 2.08) vs 1.80 (1.73, 1.82) | 2.02 (±0.049) vs 2.05 (±0.052) | 2.02 (±0.049) vs 1.88 (±0.042) | 2.04 (1.90, 2.13) vs 1.80 (1.73, 1.82) |
| p | 0.943 | 0.004 | 0.664 | 0.048 | 0.005 |
| High slope  (MPa) | 8.89 (±1.64) vs 8.85 (±1.16) | 8.89 (±1.64) vs 14.29 (±1.36) | 8.89 (±1.64) vs 8.65 (±1.07) | 8.89 (±1.64) vs 12.87 (±1.16) | 8.85 (±1.16) vs 14.29 (±1.36) |
| p | 0.984 | 0.021 | 0.905 | 0.063 | 0.007 |
|  | | | | | |
|  | ADC2/ADPC4 | ADC2/ADC5 | ADC3/ADC4 | ADC3/ADC5 | ADC4/ADC5 |
| Ultimate stress  (MPa) | 3.22 (±0.378) vs 3.10 (±0.327) | 3.22 (±0.378) vs 3.17 (±0.220) | 4.16 (±0.343) vs 3.10 (±0.327) | 4.16 (±0.343) vs 3.17 (±0.220) | 3.10 (±0.327) vs 3.17 (±0.220) |
| p | 0.806 | 0.906 | 0.038 | 0.026 | 0.857 |
| Stretch | 2.02 (±0.052) vs 2.05 (±0.052) | 2.02 (±0.052) vs 1.88 (±0.042) | 1.80 (1.73, 1.82) vs 2.06 (1.94, 2.13) | 1.80 (1.73, 1.82) vs 1.88 (1.81, 1.98) | 2.05 (±0.052) vs 1.88 (±0.042) |
| p | 0.723 | 0.048 | 0.005 | 0.096 | 0.022 |
| High slope  (MPa) | 8.85 (±1.16) vs 8.65 (±1.07) | 8.85 (±1.16) vs 12.87 (±1.16) | 14.29 (±1.36) vs 8.65 (±1.07) | 14.29 (±1.36) vs 12.87 (±1.16) | 8.65 (±1.07) vs 12.87 (±1.16) |
| p | 0.903 | 0.025 | 0.004 | 0.439 | 0.016 |
